# Machine learning assisted design of new lattice core for sandwich structures with superior load carrying capacity

**DOI:** 10.1038/s41598-021-98015-7

**Published:** 2021-09-17

**Authors:** Adithya Challapalli, Guoqiang Li

**Affiliations:** Department of Mechanical and Industrial Engineering, Louisiana State University, Baton Rouge, LA 70803 USA

**Keywords:** Engineering, Mathematics and computing

## Abstract

Herein new lattice unit cells with buckling load 261–308% higher than the classical octet unit cell were reported. Lattice structures have been widely used in sandwich structures as lightweight core. While stretching dominated and bending dominated cells such as octahedron, tetrahedron and octet have been designed for lightweight structures, it is plausible that other cells exist which might perform better than the existing counterparts. Machine learning technique was used to discover new optimal unit cells. An 8-node cube containing a maximum of 27 elements, which extended into an eightfold unit cell, was taken as representative volume element (RVE). Numerous possible unit cells within the RVE were generated using permutations and combinations through MATLAB coding. Uniaxial compression tests using ANSYS were performed to form a dataset, which was used to train machine learning algorithms and form predictive model. The model was then used to further optimize the unit cells. A total of 20 optimal symmetric unit cells were predicted which showed 51–57% higher capacity than octet cell. Particularly, if the solid rods were replaced by porous biomimetic rods, an additional 130–160% increase in buckling resistance was achieved. Sandwich structures made of these 3D printed optimal symmetric unit cells showed 13–35% higher flexural strength than octet cell cored counterpart. This study opens up new opportunities to design high-performance sandwich structures.

## Introduction

A lattice structure is formed by stacking lattice unit cells cheek by jowl in any desired order. The entire lattice structure’s performance depends on the construction of the lattice unit cell it is made of. Extensive research has been carried out in design, fabrication and evaluation of these lightweight architectures. Depending on the number of struts and number of joints in a single unit cell, it can be classified as a stretching or bending dominated structure^1^. In a stretching dominated structure, the primary mode of failure is through stretching while in a bending dominated structure the primary mode of failure is through bending. Compared to foam and bending dominated lattice structures, stretching dominated lattice structures were proven to perform better in terms of strength and stiffness^1^. Several lattice unit cells were proposed with superior performance and various advantages in structural, thermal, impact, vibrational and acoustic domains^2–6^. Octet lattice structure is one of the best truss-based lattice structures among stretching dominated lattice unit cells^3^. Gyroid and double gyroid structures manufactured through additive manufacturing exhibited decent impact absorption capabilities and additional advantage with the same stiffness in all the axial directions^4^. Hollow micro truss lattice structures were studied for their enhanced energy absorption^5^. Hybrid sandwich panels made of pyramid truss structures as core were fabricated with higher damping performance^6^. Several numerical and experimental studies were conducted by various groups to reinforce the proposed structures. Linear and nonlinear effective properties of lattice structure were studied using continuum theory models^3,7–10^. The effective properties of octet lattice structure were initially studied by Deshpande and Fleck^3^.

The fabrication techniques and structural performance of lattice cored sandwich structures were explored by different groups^11–14^. Selective laser melting (SLM) is used to manufacture bio-inspired kagome sandwich panels with titanium core. The compression and shear properties of these sandwich structures are evaluated and proved to perform better than honeycomb aerospace core structures^11^. Stereolithography (SLA) is used to print several lattice cores made of epoxy-based photopolymer resin and sandwich plates made of carbon fiber reinforced face sheets which are co-cured in two-stage manufacturing process. The compression and flexural properties of the sandwich structures were evaluated^12^. Bending response of graded lattice core sandwich structures is evaluated which achieves minimum weight sandwich structures^13^. Silicon rubber molds were used to fabricate CFRP tetrahedral core sandwich structures and tested for compression and shear strengths^14^. It is known that lattice core in sandwich structures plays an important role in the overall load carrying capacity of the sandwich. With the advancement in 3D printing, lattice core with very complex geometrical configurations can be manufactured. Therefore, further improvement in the load carrying capacity in lattice cored sandwich depends on optimization of lattice cores.

Topology optimization technique was used to further optimize existing lattice unit cells and propose unique structures. In this technique a previously available unit cell was improved iteratively for better performance by keeping the relative density constant each time^15^. Through this technique, the authors were able to develop new optimal lattice unit cells (ORC, OQSO) which were 5% and 38% stiffer compared to octet lattice unit cell in standard (001) direction^15^. To compensate for the elastic anisotropic nature of octet lattice unit cell, researchers developed elastically isotropic unit cells by merging different basic unit cells such as simple cubic unit cell, octet unit cell, etc.^16^. Optimization techniques were developed to design and automatically generate truss structures within given boundary conditions^17^. Messner^18^ proposed an inverse optimization approach and optimized an isotruss lattice, which showed about 50% increase in stiffness over the octet lattice. Yang and Li^19^ designed a cuttlebone-like lattice using topology optimization, and found that the new lattice has 141.96% increase in relative collapse strength as compared to octet lattice. Yang et al.^20^ presented a multiscale fuzzy optimization (FO) method to optimize both the density distribution and macro topology of a uniform octet-truss lattice structure. Nasrullah et al.^21^ found that octet lattice has the optimal crashworthiness, and further topology optimization found that twisted lattice structure with 20% relative density was able to generate the highest specific energy absorption. Watts^22^ used topology optimization to find maximally stiff multiscale structures comprised of micro-architected materials and find that all the trusses considered perform similarly, with anisotropic trusses resulting in slightly stiffer structures for a given load. Song et al.^23^ adopted a Kriging assisted multi-objective Genetic Algorithm to guide the design of octet-truss (OCT) cellular materials with the maximum specific modulus, and found that by optimizing the sizes of OCT, further increase in load carrying capacity can be achieved. Although classical topology optimization has been used to optimize lattice unit cell, it may be difficult to implement in designing and optimizing lattice structures with given structural boundary conditions or constraints. This is because it involves complex two-stage genetic algorithm coding. This method relies on mass reduction to design optimal structures which can overlook structures that exhibit high increase in the strength with small additions to the mass. The multiple iterations to optimize a structure and requisite to analyze the new structure for each iteration through an auxiliary software makes the process complex, extremely time consuming and necessitate high computational power, especially in the case of evaluating compound iterations. Also, through topology optimization, only a few improvised structures compared to a given reference design can be produced.

Although decent research contribution has been made in proposing novel optimal lattice unit cells, it is believed that there is a wide range of unexplored space in lattice unit cell designs that perform significantly better compared to the structures proposed so far, which calls for machine learning. Machine learning can be easily applied to scout the wide range of unexplored design space by by-passing the complex numerical analysis. The drawbacks for classical topology optimization or other optimization techniques such as time consumption, complex coding and requirement for high computational capacities, particularly for huge datasets, can be effectively compensated for through machine learning.

Auxetic metamaterials are designed using machine learning to bypass complex analytical and computational load^24^. Spinodal metamaterials with higher anisotropic stiffness are designed using neural networks and inverse design techniques^25^. Neural networks were also used to design and optimize 3D chiral metamaterial with strong chiroptical response by avoiding time consuming numerical simulations^26^. Though machine learning is used to design several metamaterials and surfaces, no study is focused on discovering new lightweight lattice truss structures and exploring the design space of such structures.

**Figure 1:**
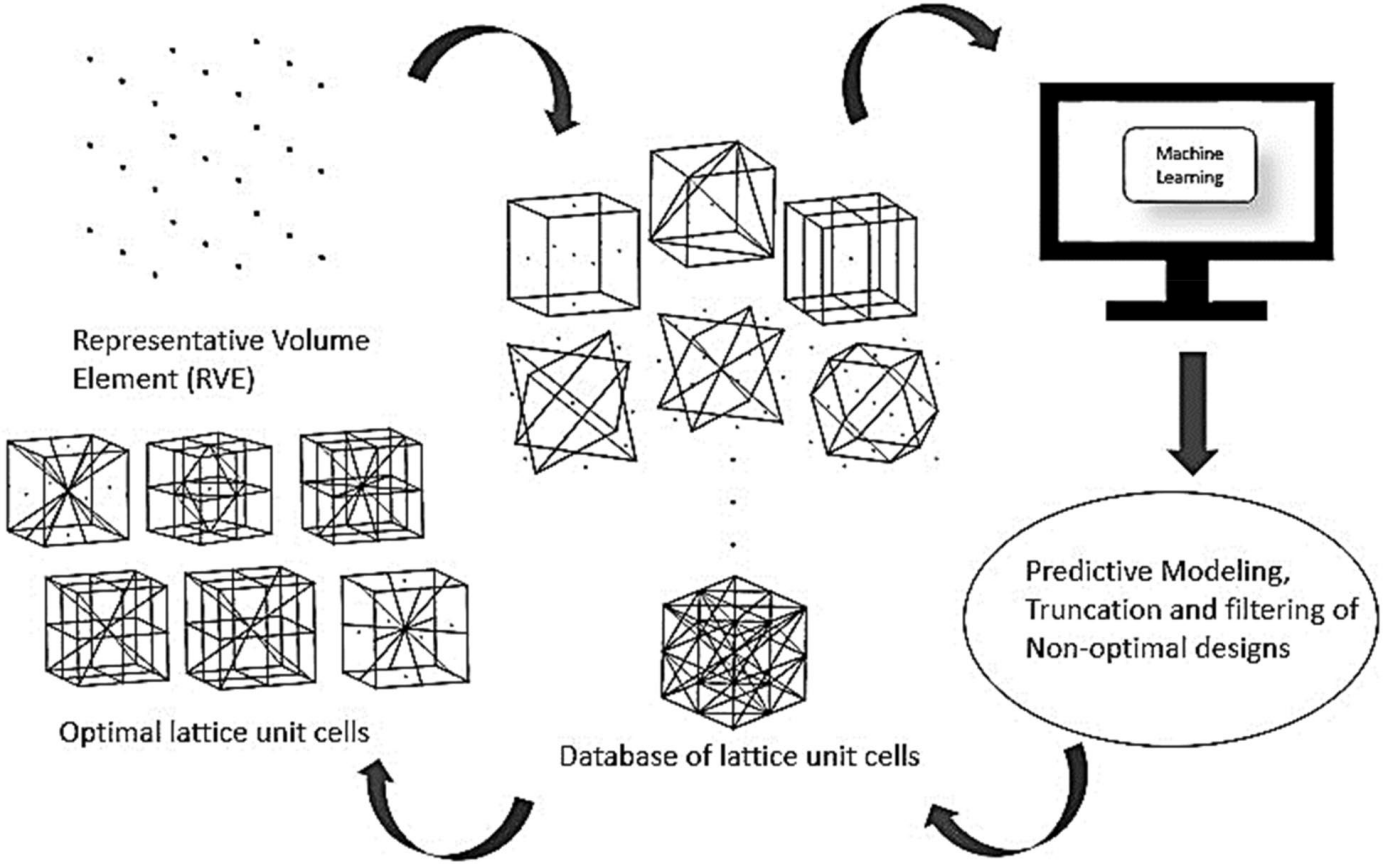
Schematic flowchart of optimization process.

The objective of this study is to design optimal stretch-dominated lattice unit cells through machine learning. To this end, this study was conducted by training an adequate portion of the generated dataset consisting of fingerprints of lattice unit cells for uniaxial compression analysis in different loading orientations. A representative volume element (RVE) made of several points was considered in this study. It was designed in such a way that a simple MATLAB code could be used to generate various possible combinations of unit lattice structures by connecting different points in the RVE. This included both stretching dominated and bending dominated lattice structures. With this, a huge dataset with combinations of various mechanisms and structures were generated. To evaluate, segregate and identify optimal structures from this ocean of data, conventional numerical and simulation methods will require enormous human power, and time and computational processing power, and still will not be optimal. To handle this issue, machine learning which is a very powerful tool in terms of computational speed and data handling can be employed. Regression training with adequate amount of input data with machine learning models like Support Vector Regression (SVR), decision trees, Gaussian Process Regression (GPR), random forest, etc., can be used to predict the stresses, deformation, and strengths in a lattice structure. Similar classification training can be used to extract several desired features from a given dataset^19^. ANSYS designer module and simulation tool were used to generate the fingerprints. This generated dataset was used to train machine learning algorithms to evaluate and predict the mechanical properties of other untrained lattice unit cells. By comparing with octet lattice unit cell, optimal lattice structures were identified (see Fig. 1 for pictorial representation). Simulation and experimental validations were conducted for uniaxial compression with respect to relative density on several optimal lattice unit cells at different orientations. Modeling conditions like the overall volume, diameter of individual truss elements, material properties, mesh sizing, boundary conditions, etc., were maintained constant for all designs for systematic comparisons. To further enhance the optimized unit cells, the solid elements in the unit cells were replaced by porous biomimetic rods, leading to significantly higher buckling resistance.

## Results and discussions

### Machine learning results

**Figure 2:**
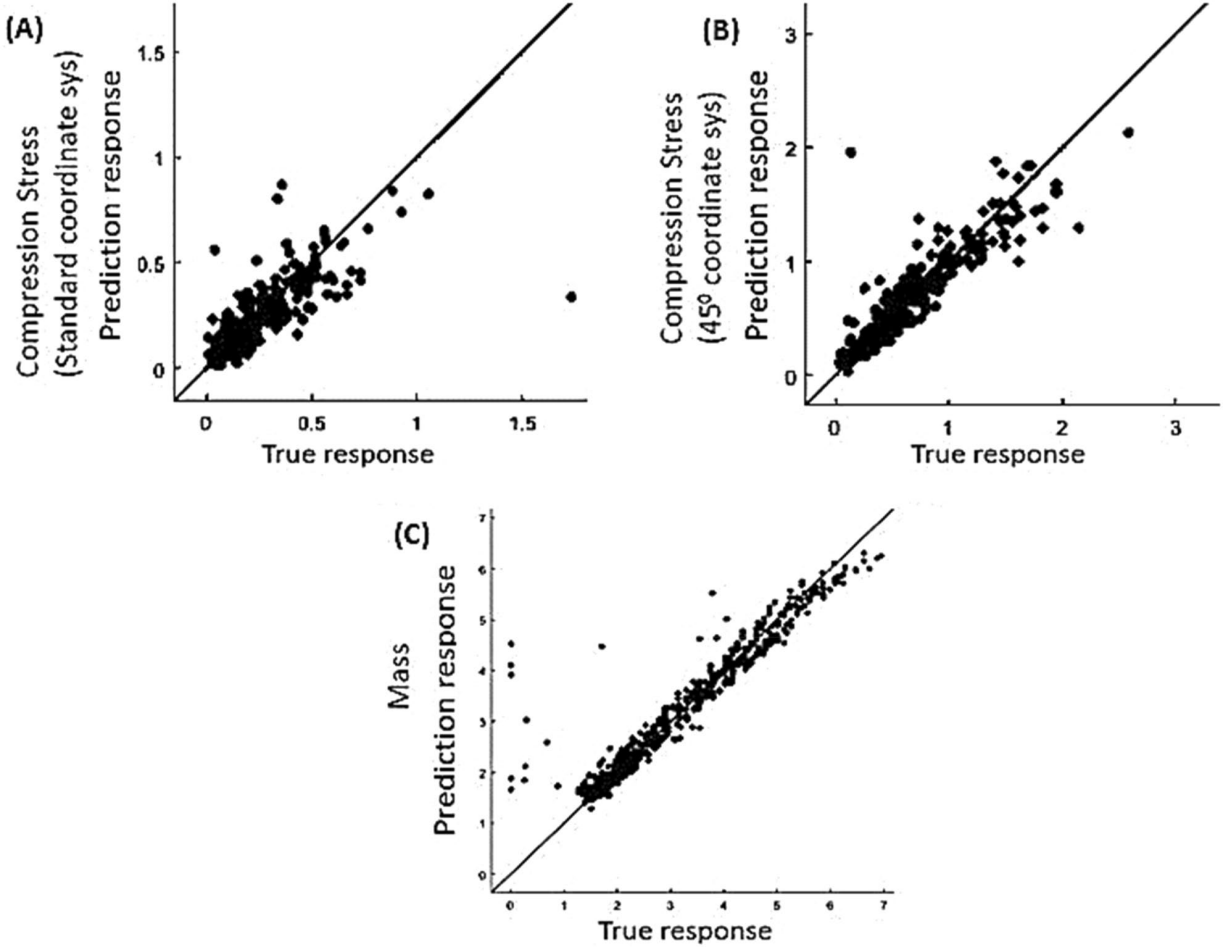
True response by ANSYS finite element analysis versus prediction by GPR machine learning algorithm for (**A**) Uniaxial compression stresses in Cartesian coordinate system, (**B**) 45° orientation compression stress, and (**C**) Mass.

Figure 2 shows the predicted vs true response plots for different properties using GPR. Clearly, the machine learning results match the true values (ANSYS simulation results) quite well with the line being predictions and the dots being the observations, the closer the observations to the prediction line, the better the model is.

Once the predictions of the mechanical properties for the dataset not used for training are accomplished using the GPR models, they are used to compare with the performance of octet lattice unit cell evaluated under the same boundary conditions. Following this scheme, a total of 20 optimal designs for symmetric lattice unit cells with superior mechanical properties over octet lattice unit cell are proposed (see S3).

### Experimental and numerical validation of the machine learning predicted unit cells

**Figure 3:**
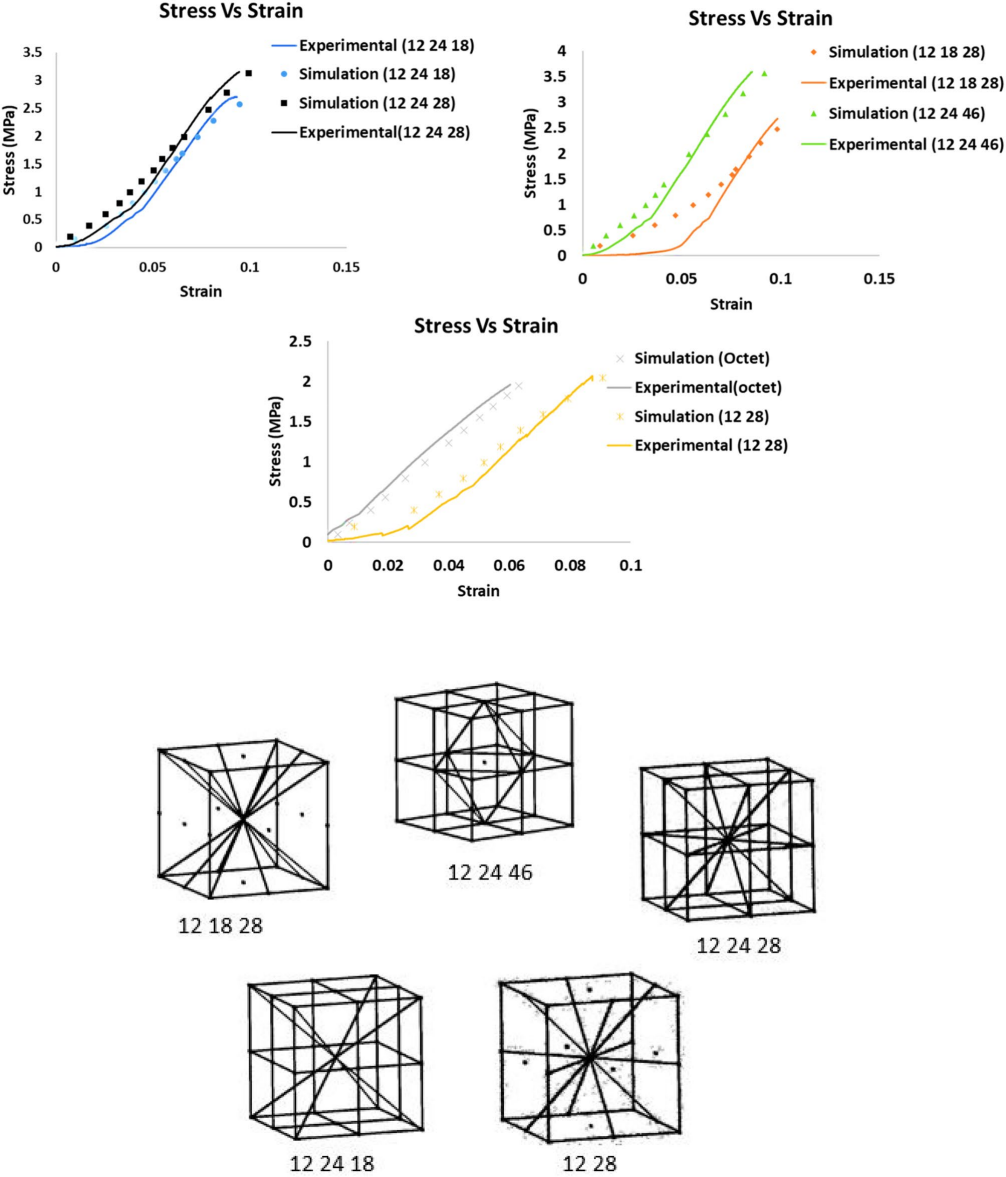
Experimental and simulation stress–strain curves of various optimal lattice unit cells along with octet truss unit cell (top), and symmetric optimal lattice unit cells with fingerprints (bottom).

A portion of these optimal lattice unit cells proposed in S3 were manufactured through 3D printing and tested under uniaxial compression. For additive manufacturing of the selected optimal lattice unit cells, Solidworks design tool is used to model and create stereolithographic (STL) files of the three-dimensional structures. These STL files can be read by any type of 3D printers. A professional 3D printer (Pico 2), which uses vat photopolymerization technique to cure materials is used for the manufacturing process. All the unit cells are manufactured using the photopolymer. Three samples of each unit cell are manufactured with varying relative densities; see images in S4. Once the postprocessing was complete, an MTS machine (ADMET eXpert 2610 Table Top 5kN Universal Test System) was used to conduct uniaxial compression test on all the samples. The compression tests were conducted at a speed of 1 mm/min and the load and displacement for each sample are recorded to get the stress–strain curves. ANSYS simulations were conducted on all the samples tested maintaining the same material properties and boundary conditions. Boundary conditions were applied on both the top and bottom surfaces of the unit cells to simulate the compression behavior. The bottom surfaces were fixed in the Z-direction, which was in the direction of the applied load, while the top surfaces on which the load was applied was allowed to freely move in the Z-direction. One of the other two directions on both the top and bottom surfaces were allowed to move to account for the effect of minor sliding. Mesh convergence was checked on the same structures used for additive manufacturing which were directly imported into the ANSYS platform for consistency (Refer to S5 for meshing and deformed shapes of the lattice unit cells). All the lattice unit cells failed through brittle facture, at low strain. Non-linearity due to imperfections in the 3D printed lattice unit cells can be observed in Fig. 3. Sample with fingerprint (12 24 46) performed better compared to the rest of 3D printed lattice unit cells in terms of compression strength. The slight variation in the experimental and simulation curves for (12 18 28) unit cells may be due to cured extra polymer resin that was not cleaned properly or left-over support protrusions. A list of more optimal lattice unit cells with both symmetric and asymmetric structures are presented in S6 and S7, respectively.

### Further simulation of machine learning predicted unit cells

**Figure 4:**
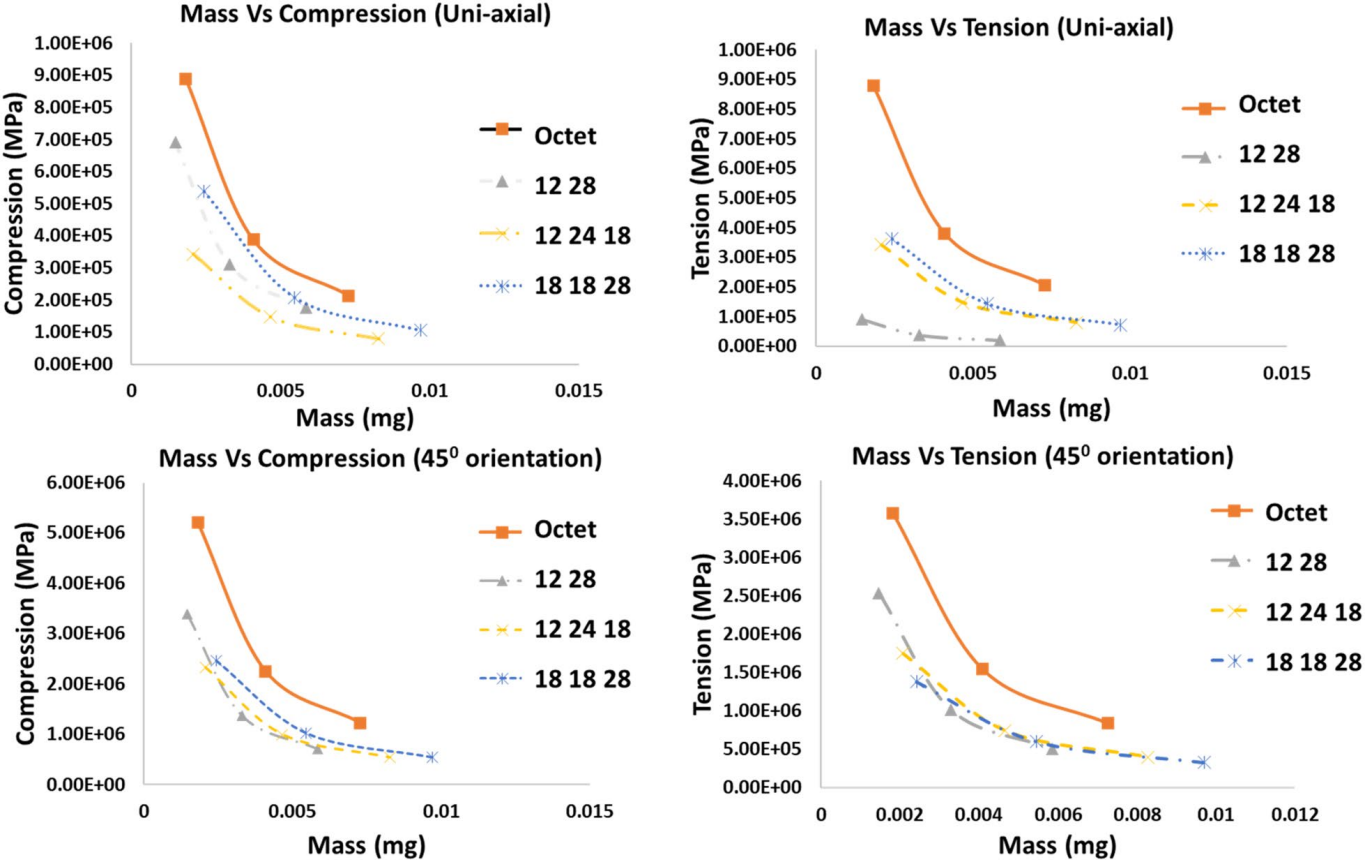
ANSYS simulation results comparisons for stresses induced in different optimal lattice unit cells compared with octet lattice unit cell.

Once the numerical results are validated by experimental results on a portion of unit cells predicted by machine learning, further simulation analysis is conducted on more optimal lattice unit cells with varying mass to observe the compression and tension stresses induced in the lattice elements under uni-axial compression in different orientations to compare with octet lattice unit cell. From Fig. 4, it can be observed that compared to octet lattice structure, the proposed optimal lattice unit cells have lower elemental compressive and tensile stresses with the same mass in normal and angular orientations. More simulation results of other optimal lattice unit cells are presented in S8.

### Three-point bending test of several lattice cored sandwich structures

**Figure 5:**
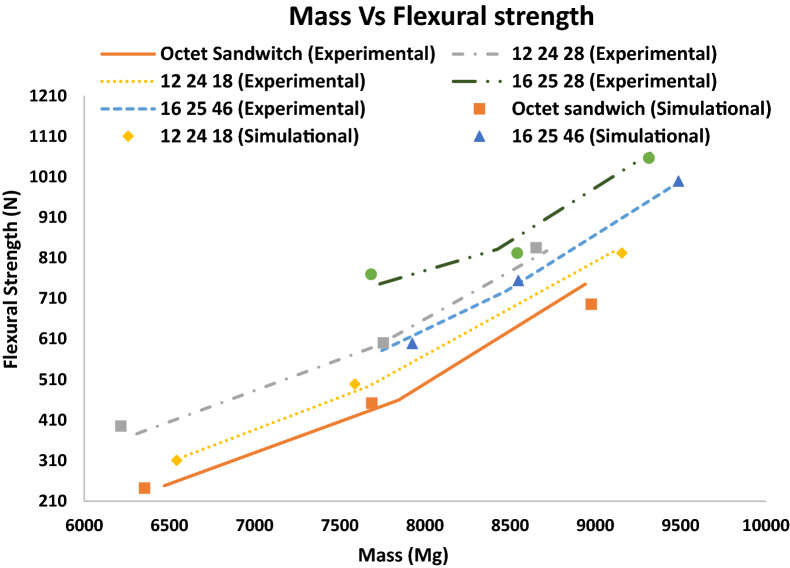
Experimental comparisons of flexural strength vs mass of various lattice cored sandwich structures made of the optimal unit cells with octet lattice cored sandwich structure.

The optimal lattice unit cells predicted by machine learning were used to design several sandwich structures with varying densities. Thin sheets with 10% of the thickness of core are used to laminate the lattice structure constructed by stacking four rows and ten columns of unit cells side by side. Three-point bending tests are performed on all the samples experimentally and compared with ANSYS simulation results. All the tests are performed on samples printed using a commercially available polymer (VeriGuide, tensile strength 28.5 MPa, elastic modulus 1.14 GPa), printed by the Pico 2 printer. After postprocessing the 3D printed parts, ADMET eXpert Test System is used to conduct the 3-point bending tests at a loading rate of 1 mm/min. For the ANSYS simulations, the support rollers are assigned to be made of steel. The contact between the support rods and sandwich structure is frictionless represented by contact interface treatment. Meshing is done using tetrahedral elements and mesh convergence is tested. Large deflection is set to “ON” to consider geometric nonlinearity. The 3-point bending test is performed by applying displacement to the top support rod and fixing the bottom two rollers. It is seen in Fig. 5 that the ANSYS simulation results are in good agreement with the experimental results. The slight difference can be attributed to the imperfection in the manufacturing and post-processing of the 3D printed sandwich structures. It can be observed from Fig. 5 that the flexural strength of sandwich structures made of the optimal symmetric unit cells is higher (13–35%) than the octet lattice sandwich structure. It is observed that sandwich structures with truss elements oriented at an angle (45°) to the sandwich plates perform better compared to sandwich panels made of elements that are perpendicular to the plates and the primary failure modes in the core are joint debonding and struct fracture. Since the entire sandwich structure is additively manufactured as a single part, no debonding of face sheets from the lattice core is observed. The images of the 3D printed sandwich structures, 3-point bending test fixture and ANSYS simulations can be found in S9.

Results of simulations for buckling capacities were also compared between the proposed optimal lattice unit cells and octet unit cell as seen in S10. It can be seen that the optimal lattice unit cells with fingerprints (12 24 48) and (12 24 46) exhibit 57% and 51% better relative buckling capacities, respectively, while fingerprints (16 25 46) and (16 24) were able to be in par with octet unit cell.

To further optimize the buckling performance of the proposed optimal lattice unit cells, the conventional solid rods (elements) of each lattice unit cell can be replaced with biomimetic rods. These biomimetic rods, inspired by the cellular structures of various plant stems, have varying outer shapes and inner porous structures. This porous nature of the biomimetic rods helps in enhancing the buckling capacity of lattice unit cells under compression^27^. Simulation analysis for the buckling capacities of optimal lattice unit cells with biomimetic rods can be seen from S11. The relative buckling capacity of the optimal lattice unit cells with biomimetic rods is 130–160% higher than that of the optimal lattice unit cells with solid rods. Images of solid designs, simulation deformation shapes and 3D printed biomimetic rods and lattice unit cells can be found in S12 and S13, respectively.

## Conclusion

Various unexplored symmetric and asymmetric isotropic and anisotropic lattice unit cells are deigned and analyzed with the help of machine learning. Machine learning helps in voluminous reduction in computational time and human effort. With proper data management and fitting training date sets, a decent estimate of structural and mechanical properties of optimal lattice unit cells are predicted. Experimental and simulation validations are conducted on selected optimal lattice unit cells. Symmetric optimal lattice unit cells designed through this approach have compression strengths from 28 to 67% higher compared to octet lattice unit cell and 13–35% in terms of flexural strength. In case of asymmetric lattice unit cells, only a portion of optimal structures are proposed in this study. To further optimize the proposed lattice unit cells, biomimetic rods were incorporated into the optimal lattice unit cells to enhance the buckling capacity of each lattice unit cell. The analysis shows that the optimized lattice unit cells with biomimetic rods achieved 130–160% increase in the relative buckling capacities as compared with the unit cells with solid rods. Depending upon the application, suitable lattice unit cells can be selected to build uniform lattice structures, sandwich panels and higher order lattice structures.

## Methods

### Dataset generation

In order to generate all possible combinations of lattice unit cells, it is essential to set certain boundary conditions for consistent execution of this approach. Here a cuboid consisting of 27 points, which can be divided into eight symmetric small cubes with eight points at the edges of each cube is considered. By connecting each point to the neighboring point, single truss elements are formed; and by combining multiple truss elements, several possible combinations of three dimensional isotropic and anisotropic lattice unit cells can be formed. One by eight of this entire cube can be considered as an RVE to generate all possible symmetric lattice unit cells (symmetric in all three standard coordinate system). This simplifies the data size, saves computer memory and reduces the complexity in handling data. The one by eight cube can be rotated in all remaining seven mirror images to build the entire lattice unit cell. Several other anisotropic combinations can be generated considering the entire cube (27 points) as the RVE to produce structures that might be optimal in direction-dependent loading conditions, both stretching and bending dominated lattice cells. When designing the training dataset using ANSYS workbench, a constant truss diameter, material parameters, meshing size and boundary conditions are set for all the fingerprints or all the lattice structures to maintain consistency. MATLAB function for enumeration of combination, “combnk” is used to generate all the possible combinations using the RVE. A total of 176 individual elements can be formed within the RVE. Several unique designs and lattice unit cells are generated covering structures consisting of from 4 elements to all the 176 elements in each structure. By doing so, nearly a million unique lattice unit cell designs can be formed, which covers a huge design space.

### Fingerprinting and machine learning

As mentioned earlier, machine learning can be used both for classification and regression. Machine learning uses the regressing tendencies from the training dataset to understand the hidden relation between different data points. Statistical models will be used to establish this relationship, which can perform the desired regression or classification exercise. Machine learning models like KRR (Kernel Ridge Regression), GPR (Gaussian process regression), neural networks (NN) and support vector machine (SVM) have been used to forward predict polymer properties based on chemical characteristics of various polymers and in discovering new polymer combinations with enhanced properties^28–30^. KRR model was used to predict the electronic dielectric, ionic dielectric and band gap properties of polymers which was used to by-pass the DFT (Density Functional Theory) calculations with average prediction error of 10% or less. Based on the polymer building block identities, fingerprints (numerical representation) of each polymer were built, which were used for the on-demand property prediction using the KRR model. Later genetic algorithm (GA) approach was used to search materials whose properties were easily predicted using the ML model, avoiding complex numerical and experimental testing^28^. Neural Networks and random forest models were trained to estimate the glass transition temperature, melting temperature and thermal conductivity of polymers. The thermal conductivity models exhibited a Mean Absolute Error (MAE) of 0.0204. Similar to the previous models, once a forward ML model is developed, a molecular design algorithm was used to generate several new samples. By using the ML models to predict the desired properties, new polymers with thermal conductivities of 0.18–0.41 W/mK were discovered^29^. SVM models were used to predict the elastic modulus of self-compacting concrete with a MAE of less than 5% as an alternative to other numerical models whose results were often ambiguous and uncertain^30^.

It can be observed from the literature that with data having logical interrelation between the predictors and the responses, machine learning tool can be more accurate and requires less data. Hence machine learning can be adequately used for the prediction of mechanical properties in structural engineering. In this study, a training dataset consisting of about two thousand random lattice unit cell models are extracted for regression training from the entire dataset generated using MATLAB. ANSYS modeling tool is used to analyze each combination. Uniaxial compression is performed with loads normal to the Cartesian coordinate axis and at angular orientations. All the models are subjected to a constant load, overall volume and material properties. While other high-performance thermoset polymers are available for 3D or 4D printing^31^, the commercially available RSI-10 photopolymer is used as the base material throughout this research. The mass, elemental compression stress, tensile stresses and deformation are recorded for each design to form a dataset for the regression training. Coherent data management is crucial for precise predictions in any machine learning process. In this study, the input data are the fingerprints of each design. Fingerprints refers to the numerical representation of the individual lattice unit cells. There is no definitive procedure in fingerprinting of the lattice unit cells for machine learning. Depending on the type of input data, fingerprints should be defined as a logical numerical sequence maintaining consistency throughout the dataset. To fingerprint each lattice unit cell in this study, each lattice point in the RVE is numbered and the element connecting any two points is fingerprinted by the numbers of the two points that the element connects. For symmetric lattice unit cells, the one eighth part of the 27-point cuboid is considered as RVE. For example (12 24 46) lattice unit cell represents three elements connecting points 1, 2 (element 12), 2, 4 (element 24) and 4, 6 (element 46) in the one eighth section. Since it is a symmetric unit cell, these three elements shall be rotated into all the remaining seven mirror planes of the 27-point cuboid to form the entire lattice unit cell. In case of asymmetric lattice unit cells, the 27-point cuboid is considered as RVE, and all the elements present in the lattice unit cell together form the fingerprint (Refer to S1 for pictorial representation). Keeping the fingerprints as the predictors (input) and desired features like mass, uniaxial compression along principal orientation and uniaxial compression with angular load orientation as response variables (outputs), different datasets are created individually. These datasets can be used to estimate the structural and mechanical properties of the reminder of the fingerprints without any further simulation or analysis. MATLAB is used to train the machine learning algorithms and predict the individual lattice unit cell characteristics. Each dataset is trained and tested with multiple machine learning algorithms to find the most suitable method. While performing machine learning, Rational Quadratic GPR (Gaussian Process Regression) produced optimal results compared to SVRs, ensemble methods and decision trees. This conclusion is made based upon the RSME (Root Mean Square Error) values of these models. A list of comparisons of several machine learning regression techniques and their performances can be found in S2. GPR is a Bayesian approach to regression which works well for small datasets^32^.

### Optimization

In order to predict lattice unit cells with optimal performance, the machine learning models developed in the previous section shall be utilized to estimate the mass and compression stress of several untrained lattice unit cell fingerprints. Using MATLAB, more than 500,000 combinations of untrained lattice unit cells are generated with the “nchoosek” function within the RVE boundary conditions. It can be understood that, although a huge dataset of lattice unit cells can be generated, not all the structures have better performance. To identify lattice unit cells with optimal performance, octet unit cell which is widely considered to have superior structural performance is considered as the datum point for comparisons. Once a dataset of several of these untrained combinations is formed, the GPR model is used to predict their mass and compression properties by using the “yfit” function in a time span of 15 min or less. The advantage of using a machine learning approach comes to play here as several lattice unit cells can be evaluated for their performance within few minutes with minimal manual effort and standard computation capacities. After predicting the desired properties, the huge datasets are truncated to only contain lattice unit cells that perform better than the octet lattice unit cell. Though the training dataset generation process requires time and manual effort, once a suitable regression model is selected, the data truncation and identification of optimal lattice unit cells can be rapid. Also, all the possible combinations within a set boundary conditions or constraints can be evaluated with minimal manual effort and time consumption. By altering the boundary conditions in the data filtering process, lattice unit cells of any desired properties can be identified. Excel data filtering or MATLAB coding can be used for topology optimization by setting any desired boundary conditions to identify optimal lattice unit cells. For example, the topology optimization can be performed by setting certain boundary conditions like low mass, high compression strength and symmetric or orthotropic truss distribution along the unit cell during the filtering process. In this study, as Octet unit cell is considered as the datum point, a filter is set to extract various fingerprints that have higher relative compression strength (with respect to the overall density) compared to the octet unit cell. Once a dataset of several optimal unit cells is obtained, more filters to extract fingerprints from the new dataset can be set. These filters include much lower mass or higher compression strength in different loading orientations and filters to extract fingerprints that have structural symmetry in the unit cells.

Although classical topology optimization can also be used to design new lattice unit cells, the technique works on material removal method from a given reference structure. Therefore, although advanced topology optimization techniques can be implemented to design complex structures, application of this technique can be complex which requires robust coding. Furthermore, classical topology optimization may be limited to a small design space, while machine learning can search optimal structures out of a large design space, and thus may predict even better structures than classical topology optimization.

## Supplementary Information

Supplementary Information.

## Data Availability

All other data are available from the authors upon reasonable request.
