# Supplementary material for: Machine learning assisted design of new lattice core for sandwich structures with superior load carrying capacity
Source: Sci Rep. 2021 Sep 17;11:18552. doi: 10.1038/s41598-021-98015-7 (PMC8448850; doi:10.1038/s41598-021-98015-7)
Supplement: Supplementary file 1 — Supplementary Information. [file 41598_2021_98015_MOESM1_ESM.docx]

**Machine Learning Assisted Design of New Lattice Core for Sandwich Structures with Superior Load Carrying Capacity**

Adithya Challapalli, Guoqiang Li

**Supplementary Information**

**
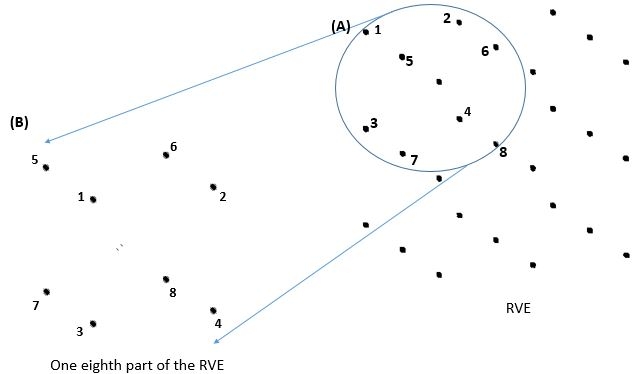
S1. Procedure to form different lattice unit cells based on representative volume element (RVE)**

**Figure S1a. (A) Representative Volume Element and (B) one eighth part of the RVE which can be used to form various combinations of symmetric lattice structures**

The one eighth part of the RVE can be used to connect different combinations of points to form all possible combinations of symmetric and asymmetric lattice unit cells. Here each point of the entire RVE is numbered from 1 to 27. One eighth part of the RVE consists of 8 points numbered from 1 to 8. Each pair of numbers represents the element connecting the respective points. To maintain consistency and meaningful connectivity, a condition is set which restricts formation of elements to connect only adjacent points, i.e., point 1 can only form an element by connecting to points 2, 3, 4, 5, 6, 7, and/or 8, which are immediately adjacent to point 1, but not with any other points from points 9 to 27 because there will be another point in between point 1 and points 9 to 27. For example (12) is an element formed by connecting point 1 and 2. Likewise all the elements in the RVE can be represented by the two points it connects. To form symmetric lattice structures, the primary and complementary elements shown in Table 1 are enough. Here the primary elements refer to the elements used to name each symmetric lattice unit cell fingerprint. For example (12 24 46) is a symmetric lattice unit cell. 12, 24 and 46 are the elements from one eighth part of the 27-point cuboid used to represent the (12 24 46) lattice unit cell as a fingerprint. To maintain symmetry, the complementary elements ((13, 15) for 12, (26, 34, 37, 56, 57) for 24, and (47, 67) for 46) attached to each primary element in Table 1 are essential. Now by using the “combnk” function, various combinations of primary elements can be generated. Combining with the complementary elements and mirror rotations, symmetric lattice truss unit cells can be designed. To form direction dependent optimal asymmetric lattice unit cells, the cuboid with 27 points is considered as RVE. Here Table 1 cannot be used as asymmetric lattice unit cells are formed by combining elements randomly within the RVE. Hence the number of elements to form a particular lattice unit cell are predefined and various combinations are generated using the same “combnk” function. For example (12 13 15 213 1314 1415 1015 910 39 519 1316 1625 1518 1827 911 1121 1922 2225 2526 2627 1920 2021 2124 2427 18 89 813 815 819 821 825 82725 23 35 28 58 46 47 67 46 68 78 45 27) is an asymmetric lattice unit cell formed by the 43 elements represented in the fingerprint. Here elements with two digits (12, 13, 15 etc.) means the element is formed by connecting two points, the first digit represents one point and the second digit represents the other point. For the elements with three digits (213, 519, 911 etc.) the first digit represents one point and second two digits are the number of other points. In case of elements with four digits (1314, 1415, 1015, etc.), the first two digits represent one point and the second two digits represent the other point forming the element. And a lattice structure or lattice core of a sandwich structure is formed by placing one of the predicted optimal unit cells side by side in rows and columns.


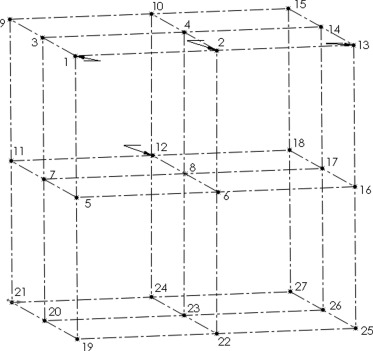
Example:

**Figure S1b. Sample cubic lattice unit cell. The vertices are named from 1 to 27 and the dotted lines connecting two adjacent vertices are the truss elements.**

Fingerprinting for lattice unit cell in figure S1b.

(12 13 15 24 26 213 37 39 34 410 414 48 56 57 519 68 616 622 711 78 812 817 823 910 911 1012 1015 1112 1121 1218 1224 1314 1316 1415 1417 1518 1625 1617 1718 1726 1827 1922 1920 2021 2023 2124 2223 2225 2324 2326 2427 2526 2627)

Here 12 refers to the truss element connecting vertices 1 and 2, similarly 13 refers to the truss element 1 and 3 and so on. The naming of the element does not depend on the sequence, i.e., the element connecting vertices 1 and can be named as 12 or 21. Since 12 does not hold any value it will be considered as a categorical variable by the machine learning algorithms.

**Table S1. Primary and their complementary elements to form symmetric lattice unit cells**

| Primary Elements | Complementary Elements | | | | |
| --- | --- | --- | --- | --- | --- |
| 12 | 13 | 15 |  |  |  |
| 16 | 14 | 17 |  |  |  |
| 24 | 34 | 37 | 56 | 57 | 26 |
| 25 | 23 | 35 |  |  |  |
| 28 | 38 | 58 |  |  |  |
| 46 | 47 | 67 |  |  |  |
| 48 | 68 | 78 |  |  |  |
| 45 | 27 | 36 |  |  |  |
| 18 |  |  |  |  |  |

**S2. Comparison of various machine learning regression model performances to predict compression and mass properties of lattice unit cells**

| Uni-axial compression (Standard coordinate system) | | | | | | |
| --- | --- | --- | --- | --- | --- | --- |
| Machine learning technique | Training dataset | | | Testing dataset | | |
|  | RMSE (Root Mean Square Error) | R^2^ | MAE (Mean Absolute Error) | RMSE (Root Mean Square Error) | R^2^ | MAE (Mean Absolute Error) |
| Rational Quadratic GPR (Gaussian Process Regression) | 0.20469 | 0.92 | 0.10348 | 0.522207 | NA | 0.436 |
| Ensemble (Bagged Tree) | 0.24138 | 0.88 | 0.11337 | 0.278609 | NA | 0.251 |
| Cubic SVM (Support Vector Machine) | 0.23717 | 0.89 | 0.13625 | 0.433322 | NA | 0.339 |
| Fine Tree | 0.33009 | 0.78 | 0.13227 | 0.522207 | NA | 0.436 |
| Uni-axial compression (45^o^ coordinate system) | | | | | | |
| Machine learning technique | Training dataset | | | Testing dataset | | |
|  | RMSE (Root Mean Square Error) | R^2^ | MAE (Mean Absolute Error) | RMSE (Root Mean Square Error) | R^2^ | MAE (Mean Absolute Error) |
| Rational Quadratic GPR (Gaussian Process Regression) | 0.26186 | 0.94 | 0.12043 | 0.2847 | NA | 0.221 |
| Ensemble (Bagged Tree) | 0.28321 | 0.93 | 0.13955 | 0.3347 | NA | 0.251 |
| Cubic SVM (Support Vector Machine) | 0.28385 | 0.93 | 0.15415 | 0.45781 | NA | 0.348 |
| Fine Tree | 0.32882 | 0.91 | 0.13205 | 0.51247 | NA | 0.412 |
| Mass | | | | | | |
| Machine learning technique | Training dataset | | | Testing dataset | | |
|  | RMSE (Root Mean Square Error) | R^2^ | MAE (Mean Absolute Error) | RMSE (Root Mean Square Error) | R^2^ | MAE (Mean Absolute Error) |
| Rational Quadratic GPR (Gaussian Process Regression) | 0.26699 | 0.97 | 0.08 | 0.118 | NA | 0.01 |
| Ensemble (Bagged Tree) | 0.38828 | 0.93 | 0.21917 | 0.00632 | NA | 0.004 |
| Cubic SVM (Support Vector Machine) | 0.70689 | 0.76 | 0.50732 | 0.459 | NA | 0.04 |
| Fine Tree | 0.45214 | 0.90 | 0.27526 | 0.305 | NA | 0.3 |

Comparison of various machine learning regression models for compression stress and mass predictions are given in the above tables. MATLAB is used to perform the regression. The built-in machine learning regression modules in MATLAB can be used to directly import the training dataset and testing dataset to the various machine learning algorithms. A five-hold cross-validation was used for all the regression models. For the Gaussian process regression and SVM models, a 300-second training time with 10 grid divisions were used. For the ensemble (bagged tree) and the fine tree models, the minimum leaf size of 8 and 30 learners were used with a learning rate of 0.1. The RSME, R-squared and MAE values in the above tables are available with each training algorithm in the MATLAB regression learner application. For the testing dataset, the RMSE and MAE are calculated using the following formula:

$$RMSE=\sqrt{\left( \frac{\sum_{i=1}^{N} (x_{i}-y_{i})^{2}}{N} \right)}$$

$$MAE=\frac{\sum_{i=1}^{N} \left| y_{i}-x_{i} \right|}{N}$$

where

RMSE = Root Mean Square Error;

MAE = Mean Absolute Error;

$x_{i}$ = true values;

$y_{i}$ = predicted values;

$N$ = Total number of observations;

$i$ = variable i;

**S3. Selected optimal lattice unit cells**

| Unit cells | Fingerprints | Unit cells | Fingerptins | Unit cells | Fingerprints |
| --- | --- | --- | --- | --- | --- |
| 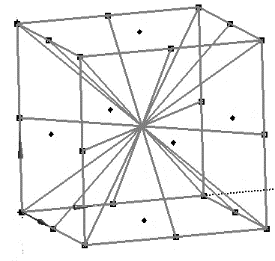 | 12 18 28 | 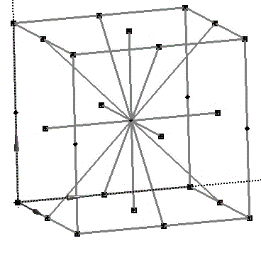 | 12 28 48 | 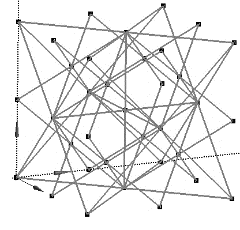 | 16 45 48 |
| 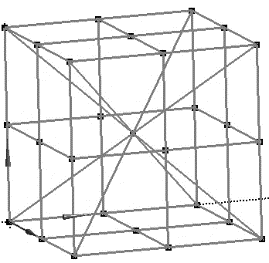 | 12 18 24 | 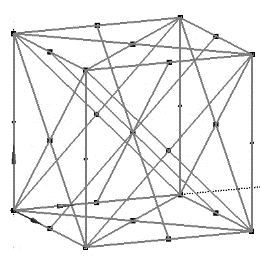 | 12 16 28 | 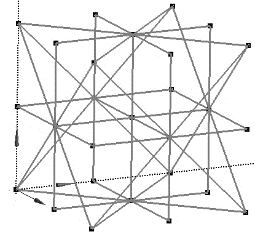 | 16 24 48 |
| 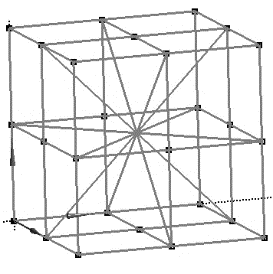 | 12 24 28 | 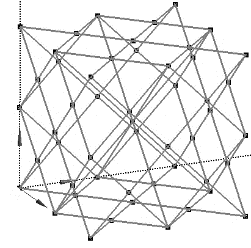 | 16 25 28 | 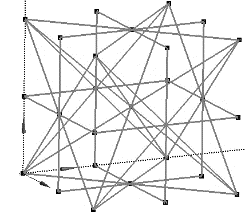 | 16 24 18 |
| 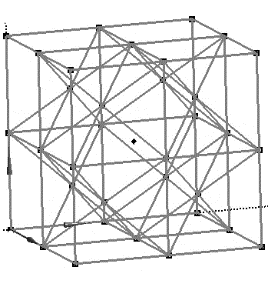 | 12 24 45 | 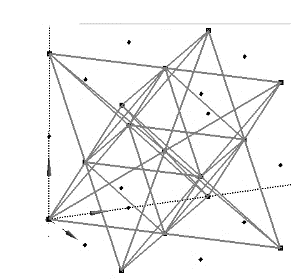 | 16 18 46 | 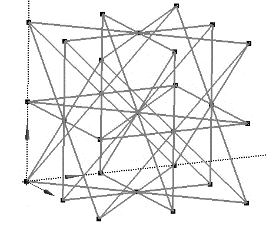 | 16 24 28 |
| 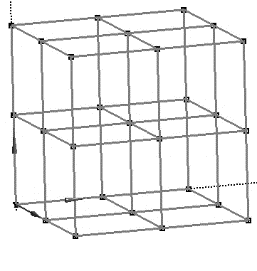 | 12 24 48 | 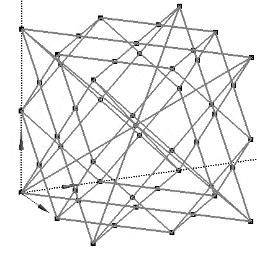 | 16 18 25 | 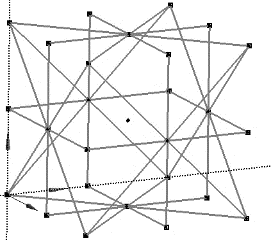 | 16 24 |
| 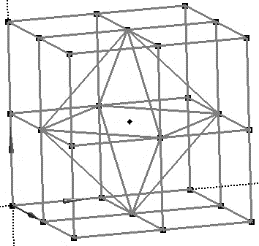 | 12 24 46 | 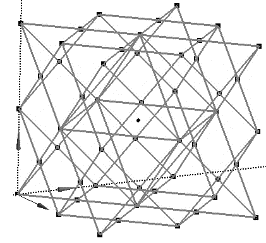 | 16 25 46 | 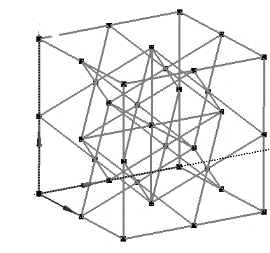 | 12 48 45 |
| 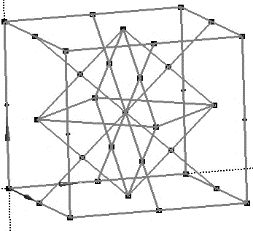 | 12 846 | 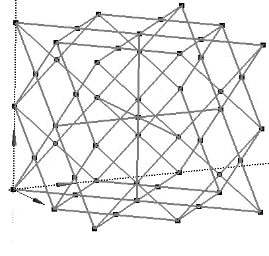 | 16 25 48 |  |  |

**S4. 3D printed unit cells**


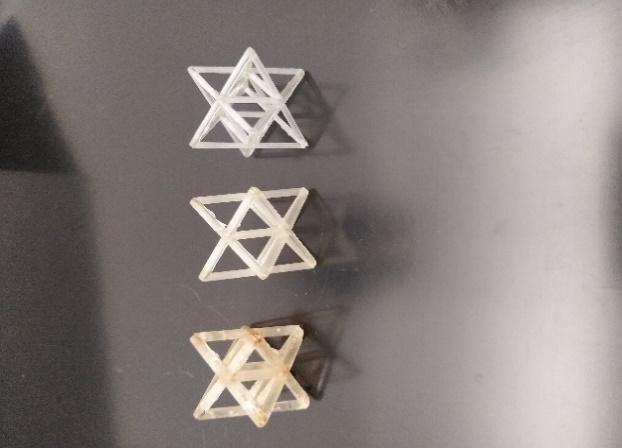

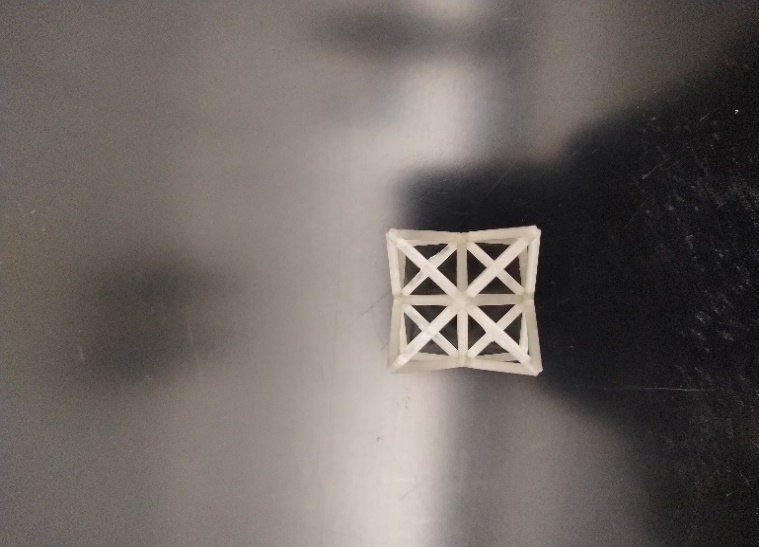

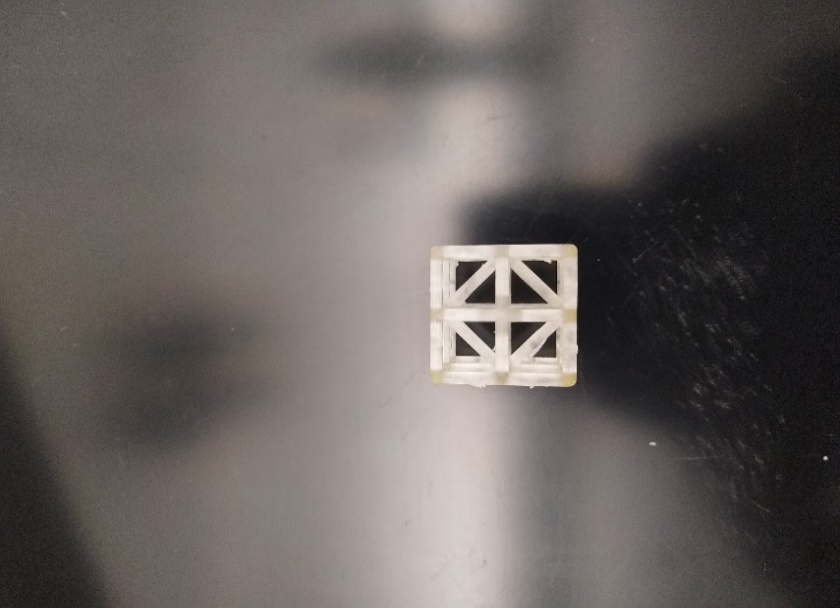

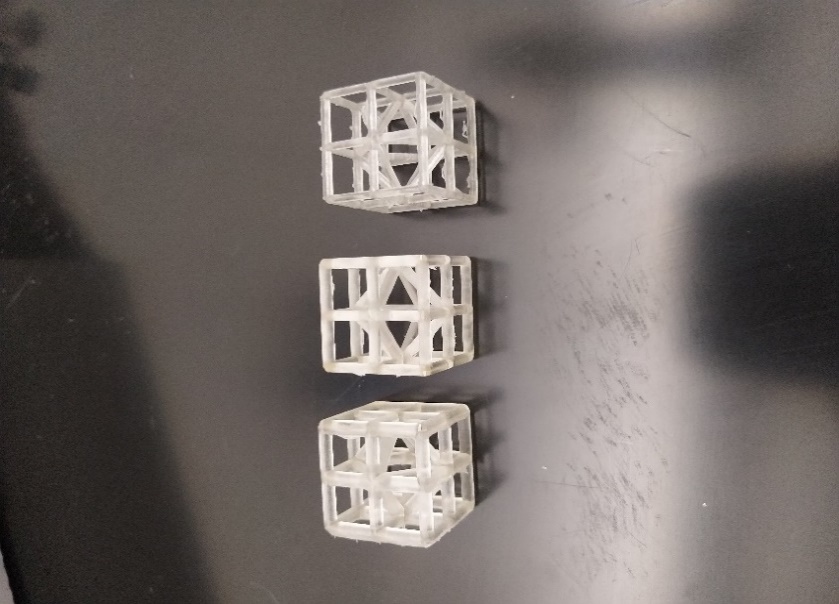

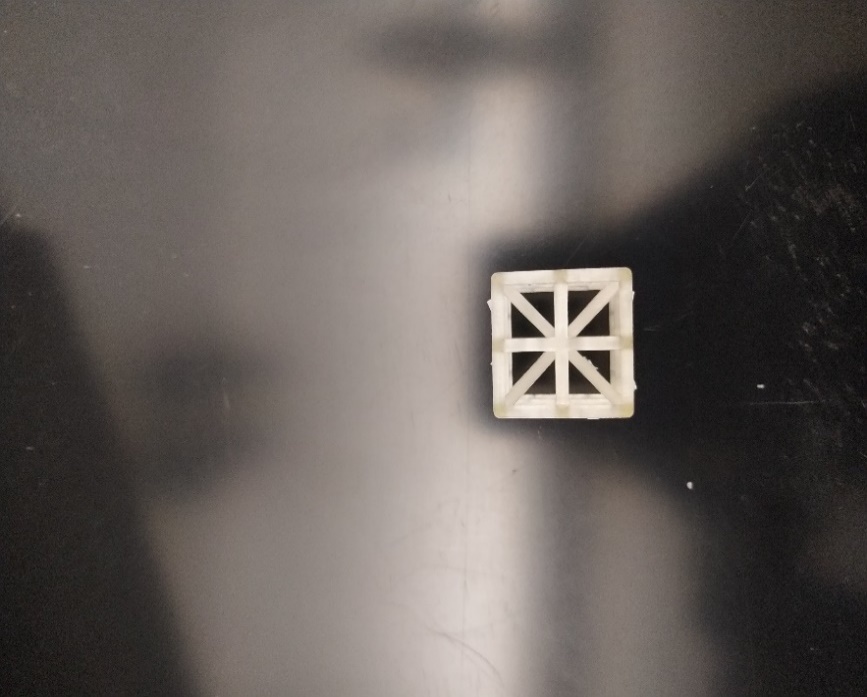

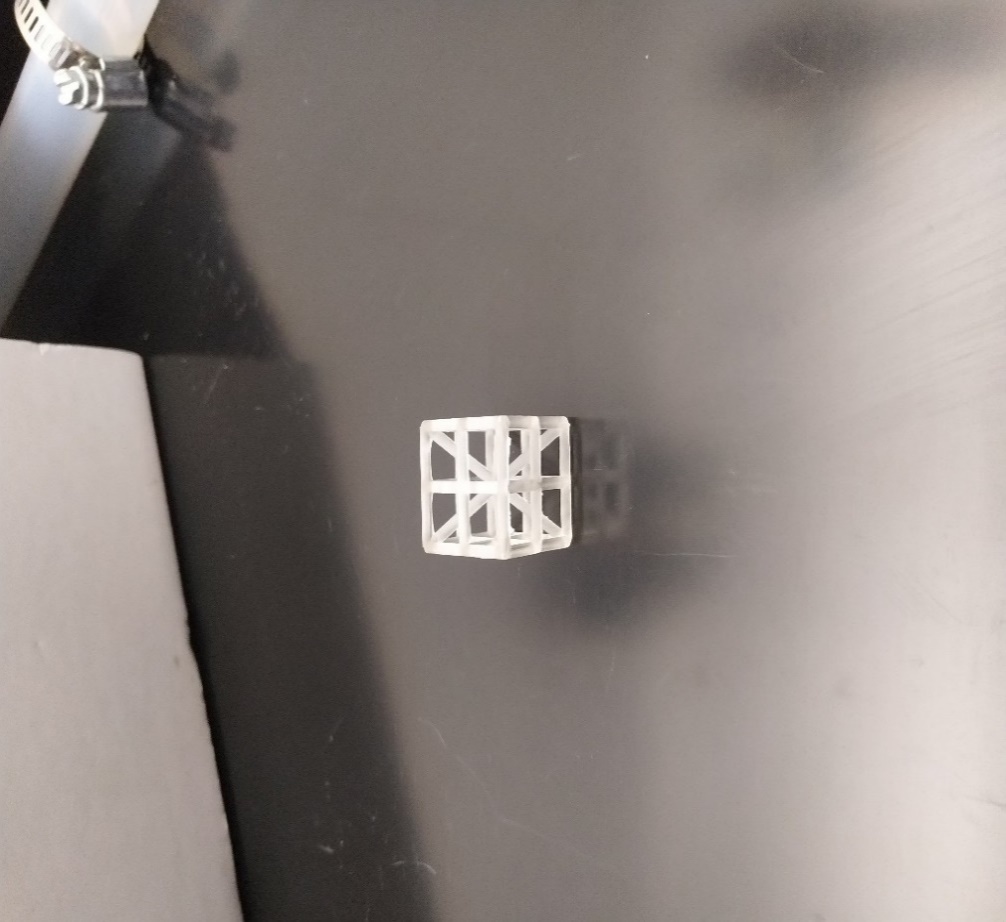

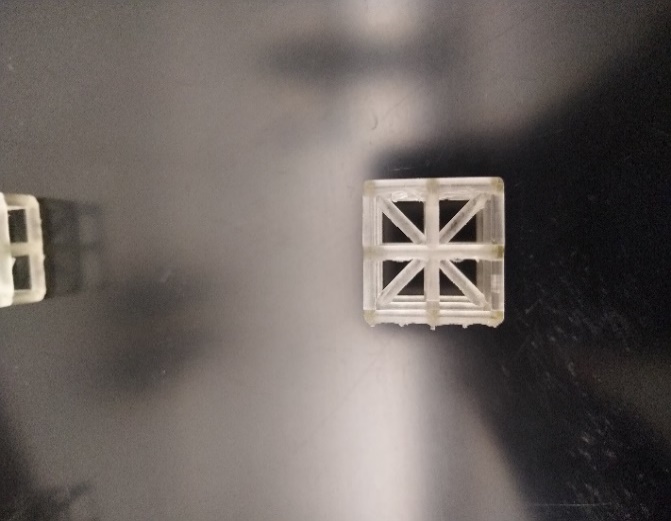

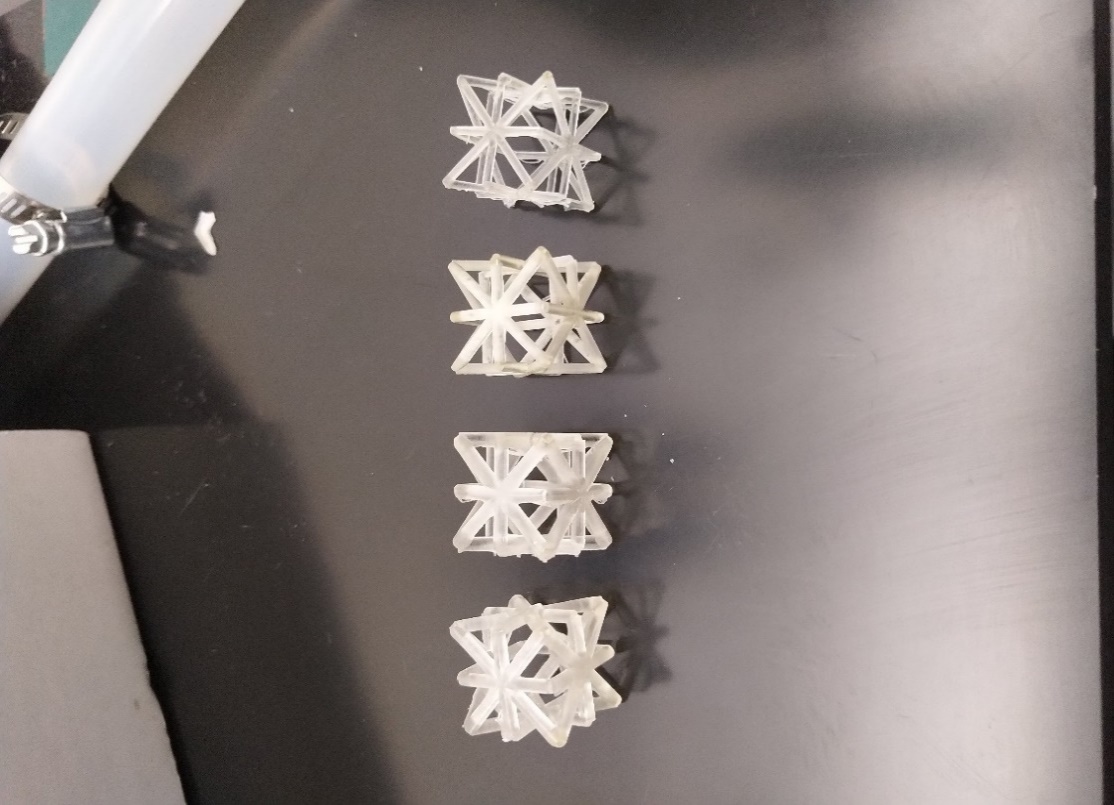

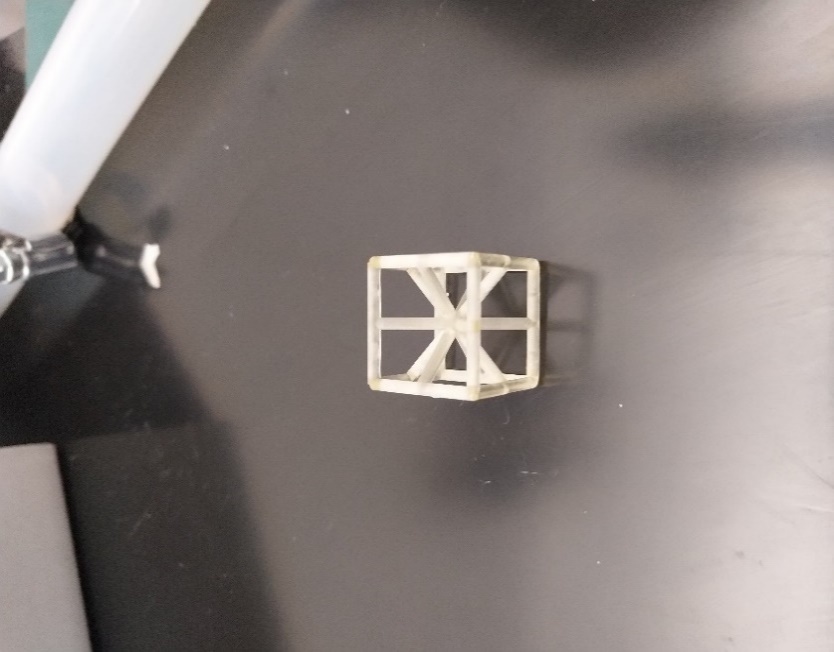

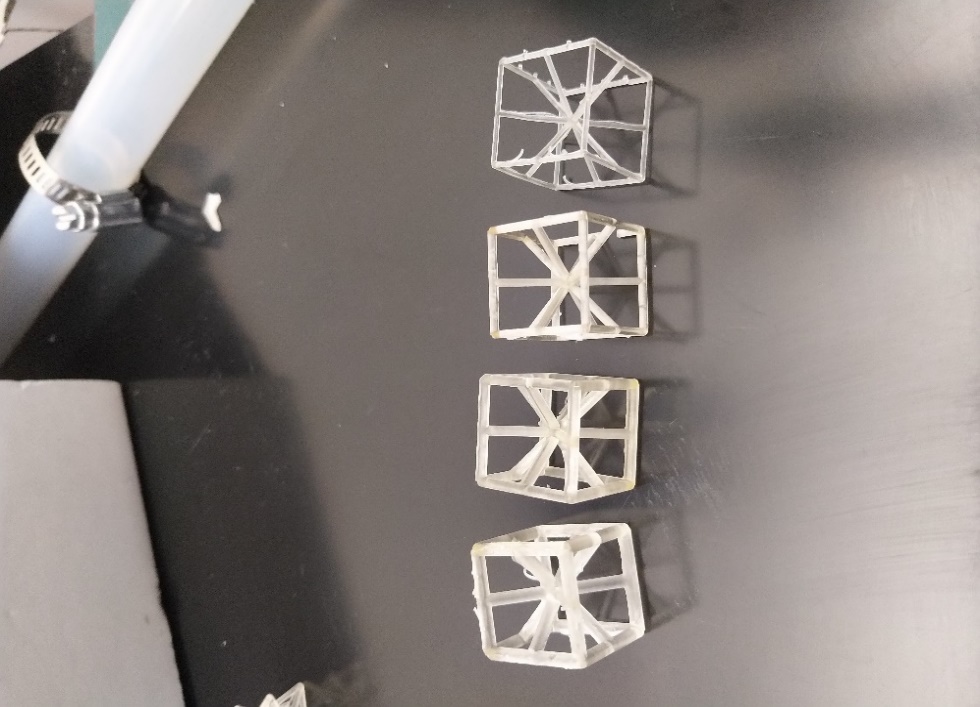

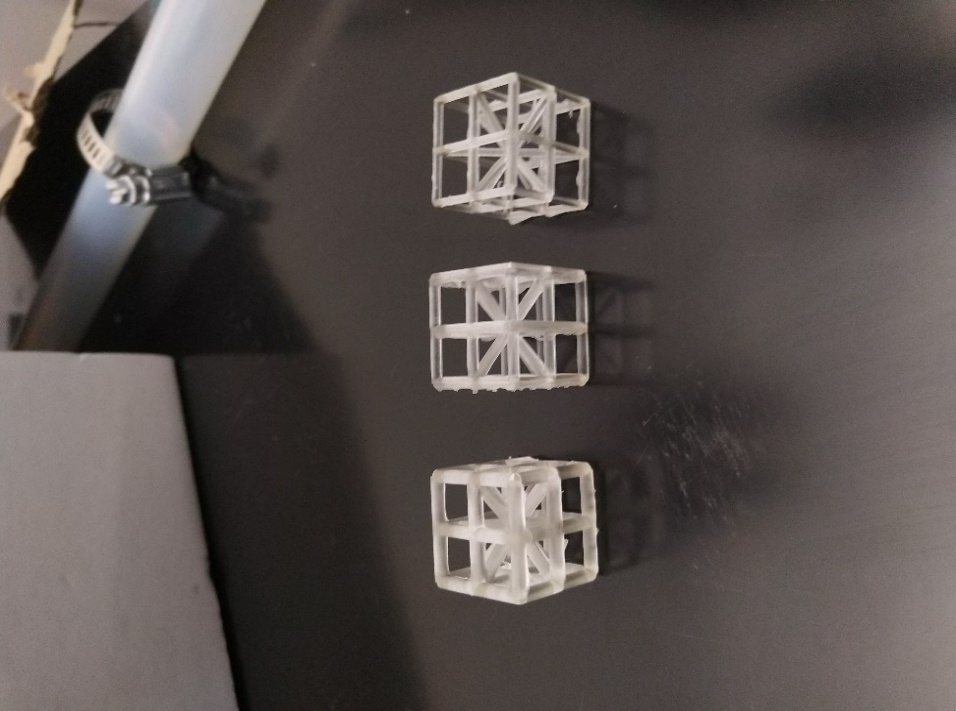

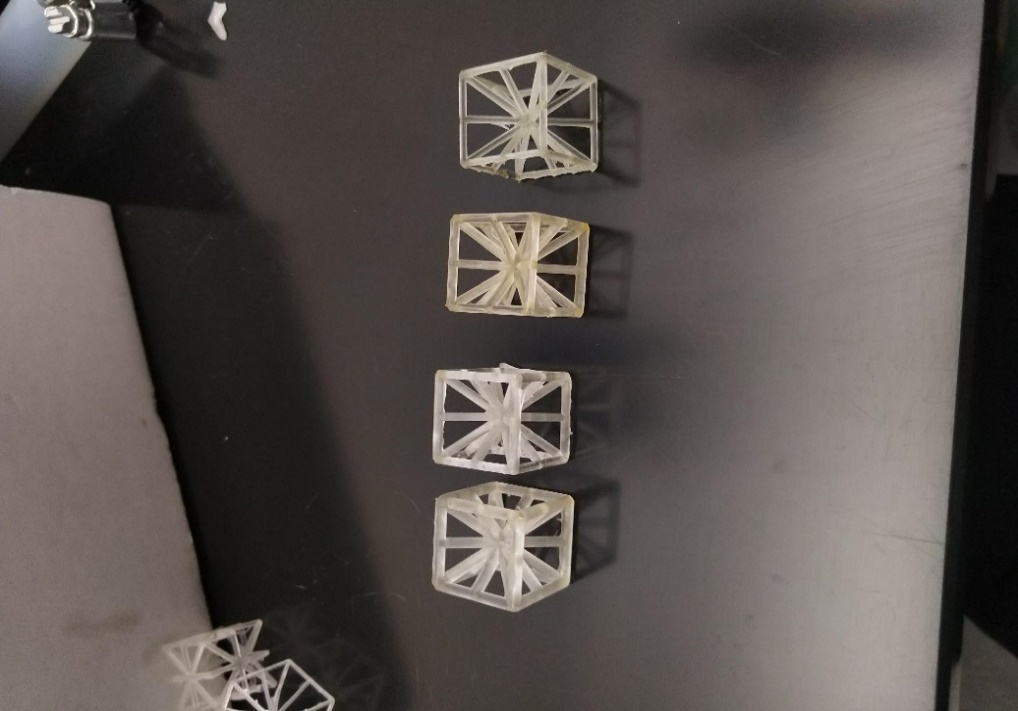

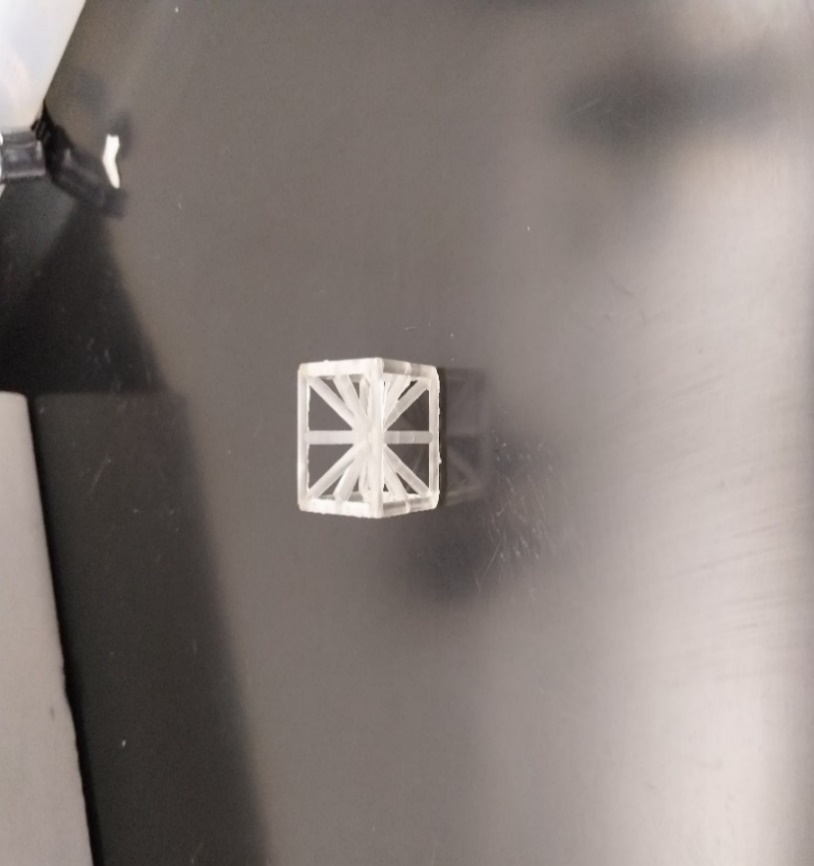

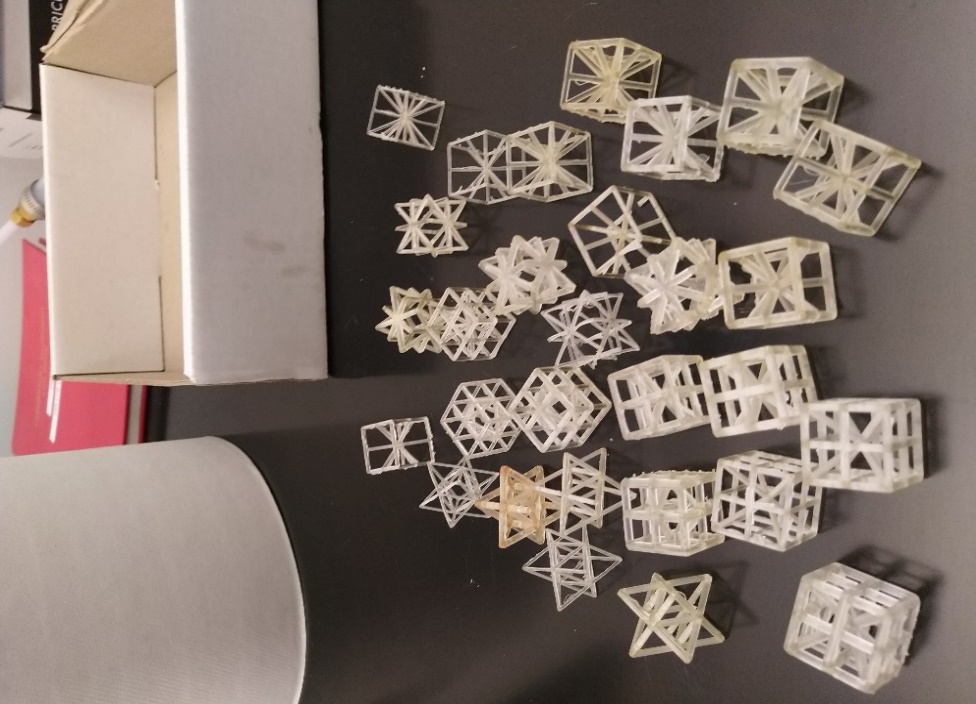


(A)

(B)

(F)

(E)

(C)

(D)

(H)

(G)

**Figure S2. Images of various 3D printed lattice unit cells (A) Octet, (B) 12 24 46, (C) 12 24 48, (D) 12 24 28, (E) 24 14, (F) 12 18 28, (G) 12 28, and (H) Different 3D printed lattice structures.**

**
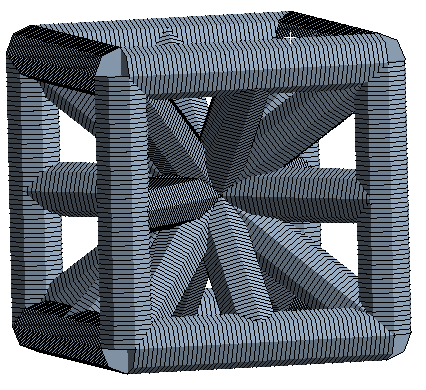

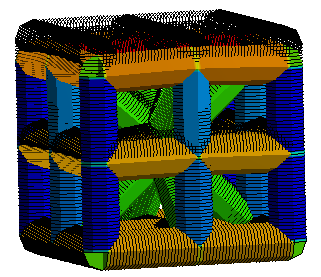

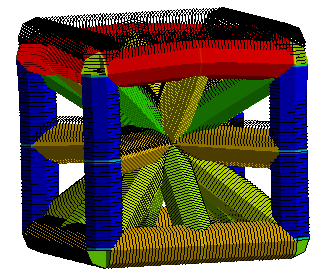

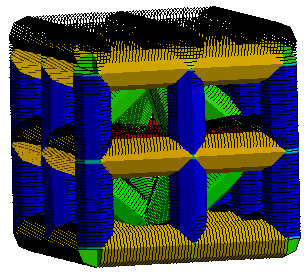

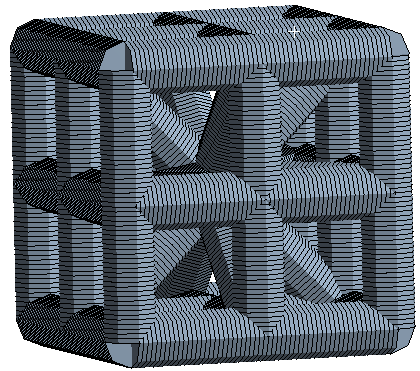

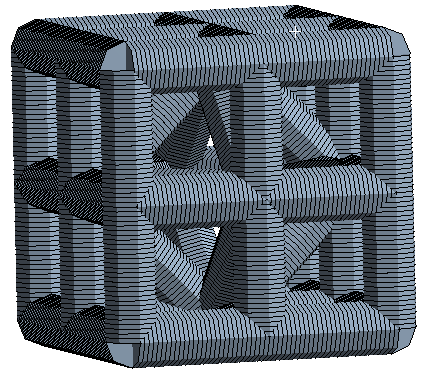
S5. ANSYS simulations for lattice unit cells**

**Figure S3. ANSYS mesh and deformation contours of few lattice unit cells.**

**S6. Complete list of optimal symmetric lattice unit cells proposed through machine learning**

| 12 28 |
| --- |
| 16 24 |
| 16 25 |
| 16 45 |
| 12 16 25 |
| 12 16 28 |
| 12 16 45 |
| 12 18 24 |
| 12 18 28 |
| 12 18 45 |
| 12 24 25 |
| 12 24 28 |
| 12 24 46 |
| 12 24 48 |
| 12 25 28 |
| 12 28 48 |
| 12 46 45 |
| 12 48 45 |
| 16 18 24 |
| 16 18 25 |
| 16 18 46 |
| 16 18 45 |
| 16 24 25 |
| 16 24 28 |
| 16 24 46 |
| 16 24 48 |
| 16 24 45 |
| 16 25 28 |
| 16 25 46 |
| 16 25 48 |
| 16 25 45 |
| 16 28 46 |
| 16 28 45 |
| 16 46 45 |
| 16 48 45 |

**S7. Sample list of optimal asymmetric lattice unit cells proposed through machine learning**

| Asymmetric optimal lattice unit cell in normal direction (uni-directional) |
| --- |
| 12 13 15 213 1314 1415 1015 910 39 519 1316 1625 1518 1827 911 1121 1922 2225 2526 2627 1920 2021 2124 2427 18 89 813 815 819 821 825 82725 23 35 28 58 46 47 67 46 68 78 45 27 |
| 12 13 15 213 1314 1415 1015 910 39 519 1316 1625 1518 1827 911 1121 1922 2225 2526 2627 1920 2021 2124 2427 24 34 37 56 57 26 25 23 35 28 38 58 46 68 |
| 12 13 15 213 1314 1415 1015 910 39 519 1316 1625 1518 1827 911 1121 1922 2225 2526 2627 1920 2021 2124 242718 89 813 815 819 821 825 82724 34 37 26 25 23 35 28 38 58 46 47 67 46 68 78 45 |
| 12 13 15 213 1314 1415 1015 910 39 519 1316 1625 1518 1827 911 1121 1922 2225 2526 2627 1920 2021 2124 2427 18 89 813 815 819 821 825 82724 34 37 56 57 26 25 23 35 28 38 58 46 47 |
| 12 13 15 213 1314 1415 1015 910 39 519 1316 1625 1518 1827 911 1121 1922 2225 2526 2627 1920 2021 2124 2427 24 34 37 56 57 26 25 23 35 28 38 58 46 68 78 45 27 |
| 12 13 15 213 1314 1415 1015 910 39 519 1316 1625 1518 1827 911 1121 1922 2225 2526 2627 1920 2021 2124 242718 89 813 815 819 821 825 82725 23 35 46 47 67 46 68 78 45 27 36 |
| 12 13 15 213 1314 1415 1015 910 39 519 1316 1625 1518 1827 911 1121 1922 2225 2526 2627 1920 2021 2124 2427 25 23 35 28 46 47 67 46 68 78 45 27 36 |
| 12 13 15 213 1314 1415 1015 910 39 519 1316 1625 1518 1827 911 1121 1922 2225 2526 2627 1920 2021 2124 2427 24 34 37 56 57 26 28 67 46 68 78 45 27 36 |
| 12 13 15 213 1314 1415 1015 910 39 519 1316 1625 1518 1827 911 1121 1922 2225 2526 2627 1920 2021 2124 2427 24 34 37 56 57 26 25 68 78 45 27 36 |
| 12 13 15 213 1314 1415 1015 910 39 519 1316 1625 1518 1827 911 1121 1922 2225 2526 2627 1920 2021 2124 2427 24 34 37 56 57 26 25 23 35 28 38 58 78 45 27 36 |
| 16 14 17 625 613 619 912 1227 1215 1221 79 719 721 49 413 415 1317 1517 1725 1727 1923 2123 2325 2327 18 89 813 815 819 821 825 82724 34 37 56 57 35 28 38 58 46 68 78 45 27 36 |
| Asymmetric optimal lattice unit cell in 45^0^ orientation direction (uni-directional) |
| 16 14 17 625 613 619 912 1227 1215 1221 79 719 721 49 413 415 1317 1517 1725 1727 1923 2123 2325 2327 18 89 813 815 819 821 825 82724 34 37 56 57 26 25 23 35 28 45 27 36 |
| 16 14 17 625 613 619 912 1227 1215 1221 79 719 721 49 413 415 1317 1517 1725 1727 1923 2123 2325 2327 18 89 813 815 819 821 825 82724 34 37 56 57 26 38 58 46 47 67 46 68 78 |
| 16 14 17 625 613 619 912 1227 1215 1221 79 719 721 49 413 415 1317 1517 1725 1727 1923 2123 2325 2327 18 89 813 815 819 821 825 82724 34 37 56 57 26 25 23 35 48 68 36 |
| 16 14 17 625 613 619 912 1227 1215 1221 79 719 721 49 413 415 1317 1517 1725 1727 1923 2123 2325 2327 625 613 619 912 1227 1215 1221 79 719 721 49 413 415 1317 1517 1725 1727 1923 2123 2325 2327 56 57 26 25 23 35 46 47 67 45 27 36 |
| 16 14 17 625 613 619 912 1227 1215 1221 79 719 721 49 413 415 1317 1517 1725 1727 1923 2123 2325 2327 18 89 813 815 819 821 825 82724 34 26 25 23 35 46 47 67 46 68 78 |
| 16 14 17 625 613 619 912 1227 1215 1221 79 719 721 49 413 415 1317 1517 1725 1727 1923 2123 2325 2327 18 89 813 815 819 821 825 82724 34 37 56 57 26 28 38 58 45 27 36 |
| 16 14 17 625 613 619 912 1227 1215 1221 79 719 721 49 413 415 1317 1517 1725 1727 1923 2123 2325 2327 18 89 813 815 819 821 825 82724 34 37 56 57 26 25 23 35 28 38 78 |
| 16 14 17 625 613 619 912 1227 1215 1221 79 719 721 49 413 415 1317 1517 1725 1727 1923 2123 2325 2327 18 89 813 815 819 821 825 82724 34 37 56 57 26 38 58 46 47 67 |
| 16 14 17 625 613 619 912 1227 1215 1221 79 719 721 49 413 415 1317 1517 1725 1727 1923 2123 2325 2327 18 89 813 815 819 821 825 82724 34 37 56 57 26 25 23 35 28 38 58 46 68 78 45 |

**S8. Modeling results comparison**

**
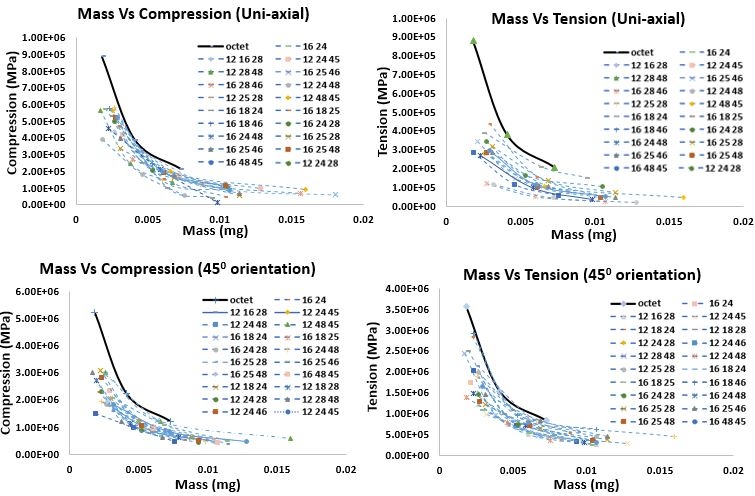
**

**Figure S4. Several optimal symmetric lattice truss unit cells compared to octet truss under uniaxial compression in axial and angular orientation**

**S9. Images of the 3D printed sandwich structures and 3-point bending test fixture**


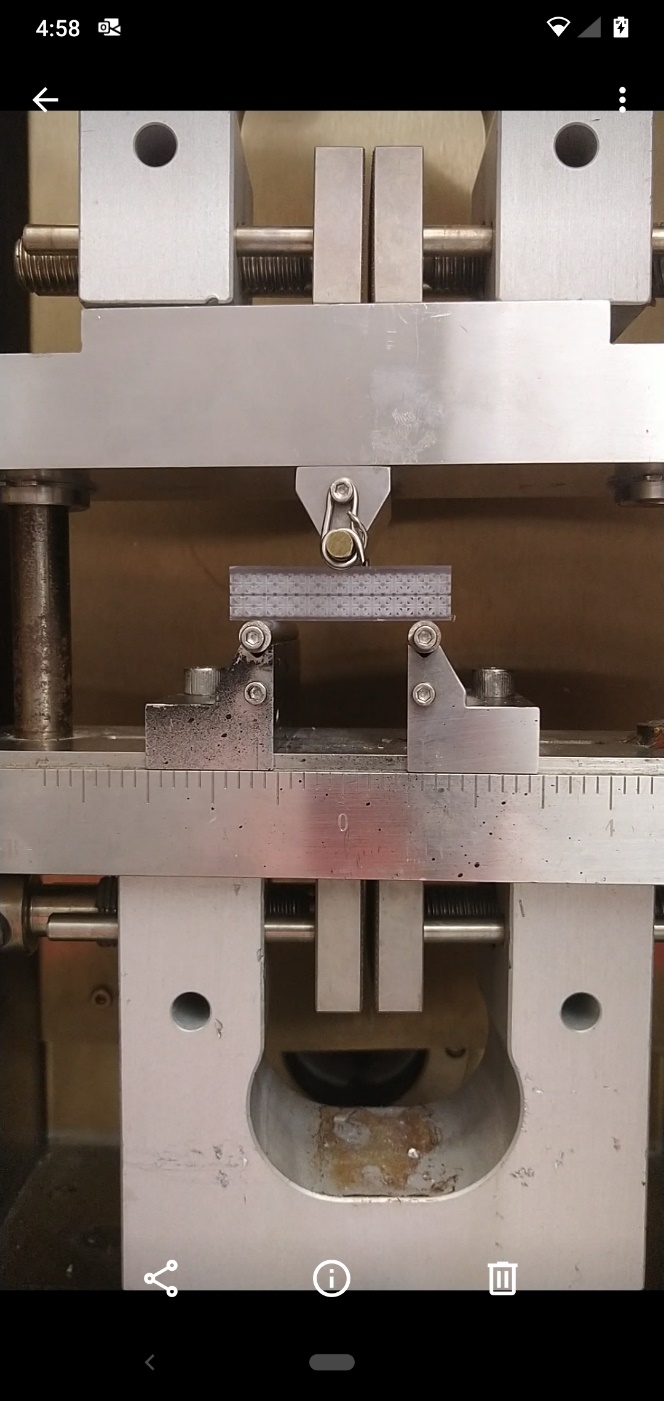

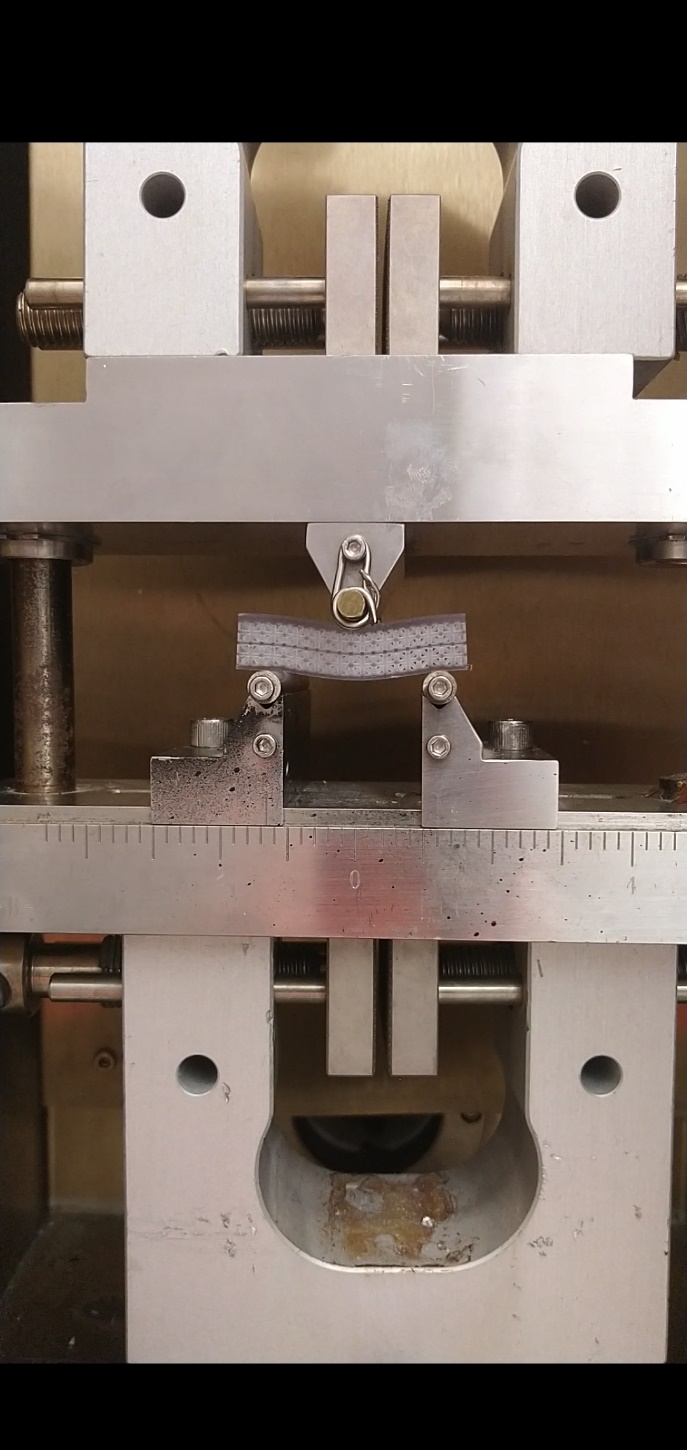
 (A)


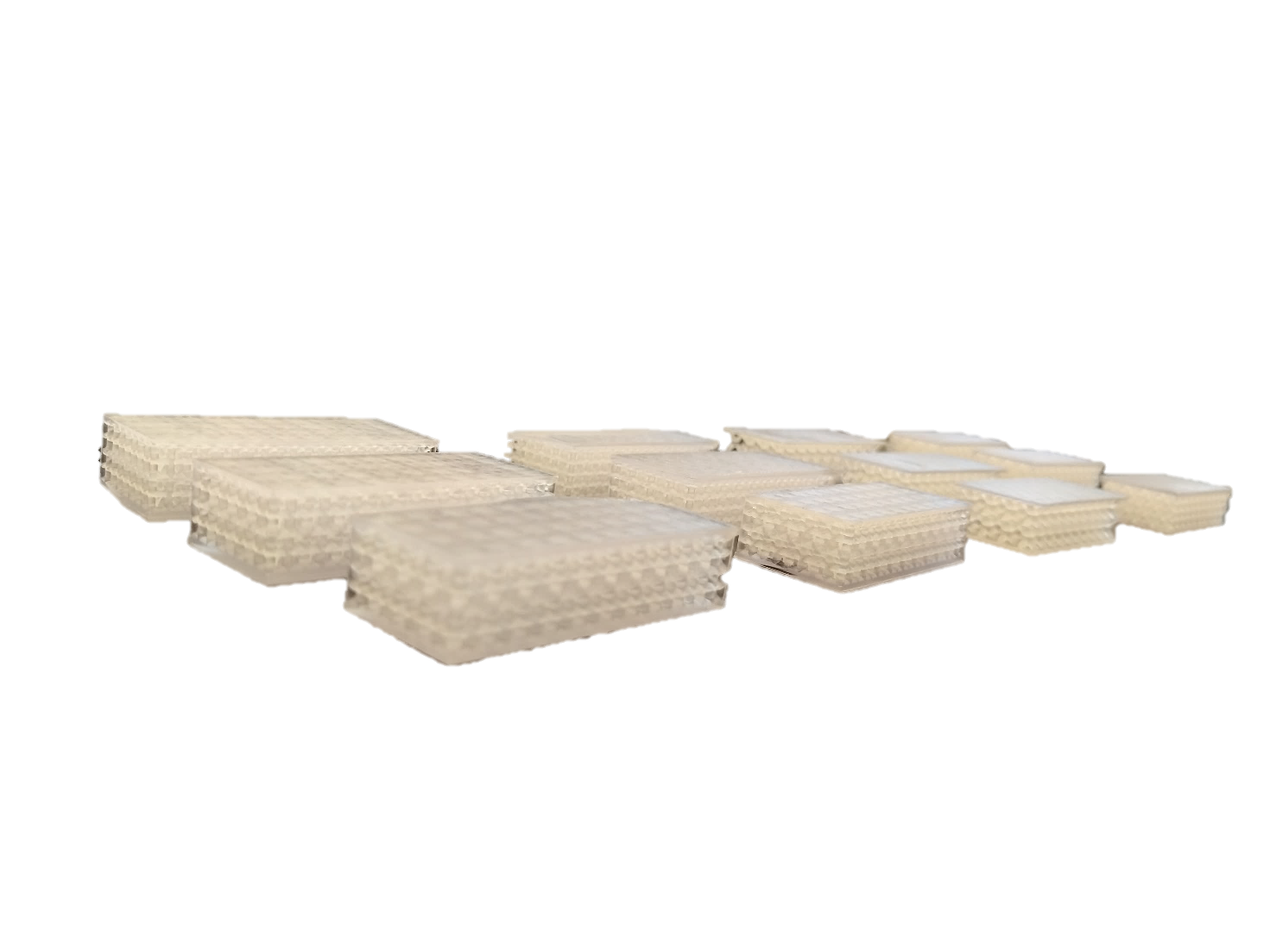


(B)

(C)


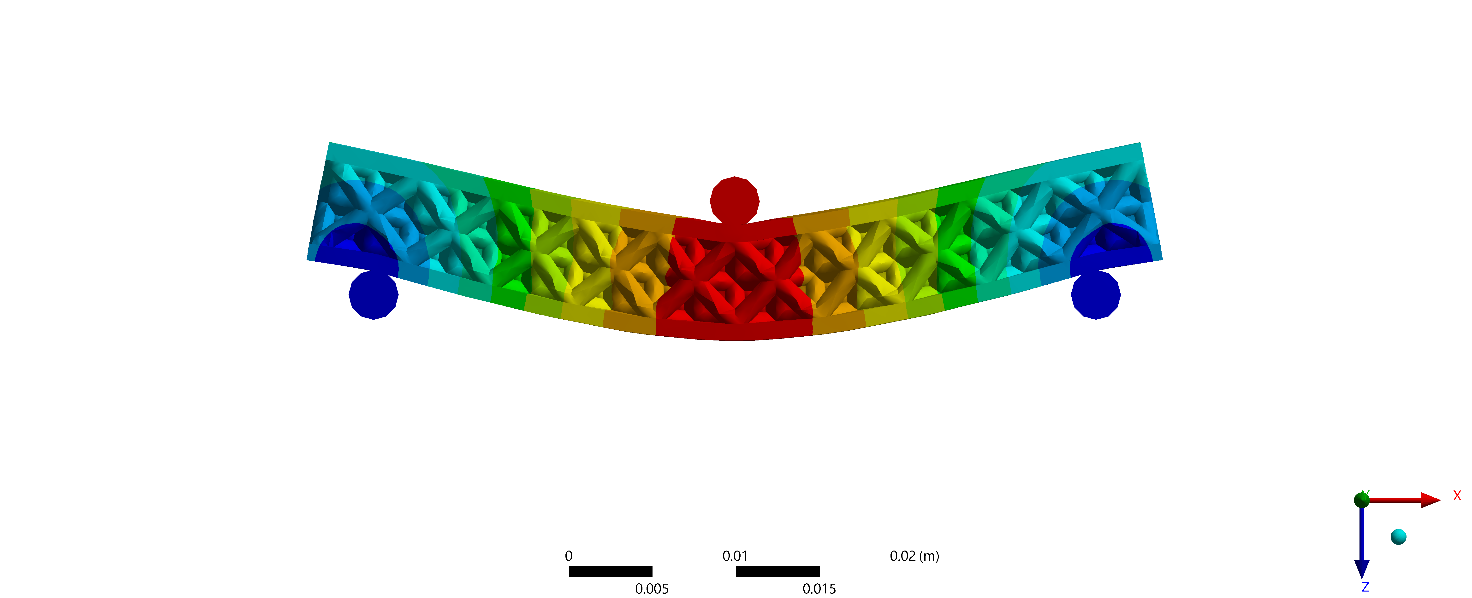


**Figure S5. (A) Several lattice 3d printed sandwich structures, (B) A sandwich structure with no applied load (left) and just before fracture (right), (C) ANSYS simulation**

**S10. Simulation of machine learning predicted unit cells with biomimetic rods**


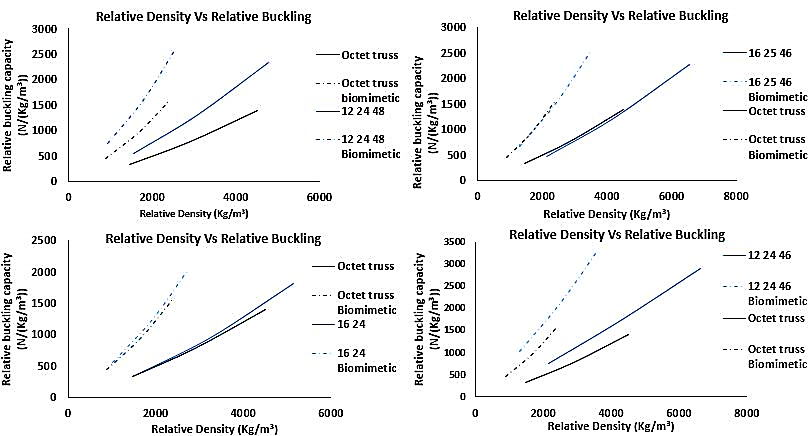


**Figure S6. ANSYS comparisons for relative buckling capacity of various optimal lattice unit cells compared with octet unit cell.**

**
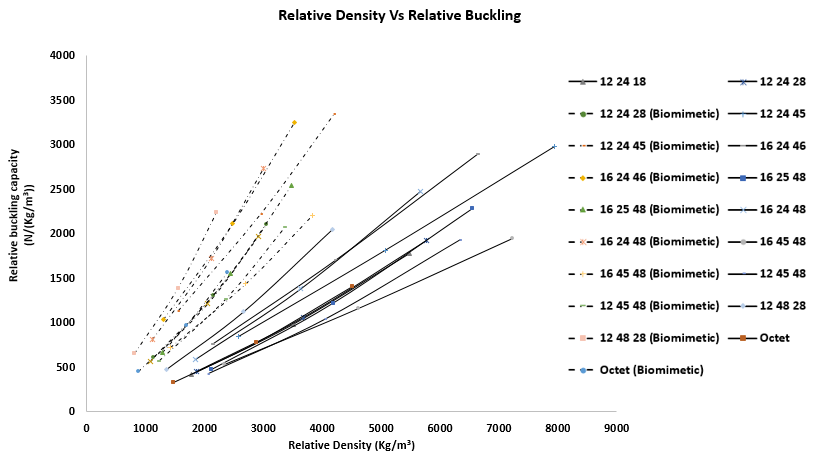
S11. Modeling results for buckling of lattice unit cells with biomimetic rods and solid rods**

**Figure S7. Buckling analysis of optimal lattice unit cells with biomimetic rods and (dotted curves) and solid cylindrical rods (solid curves).**

**
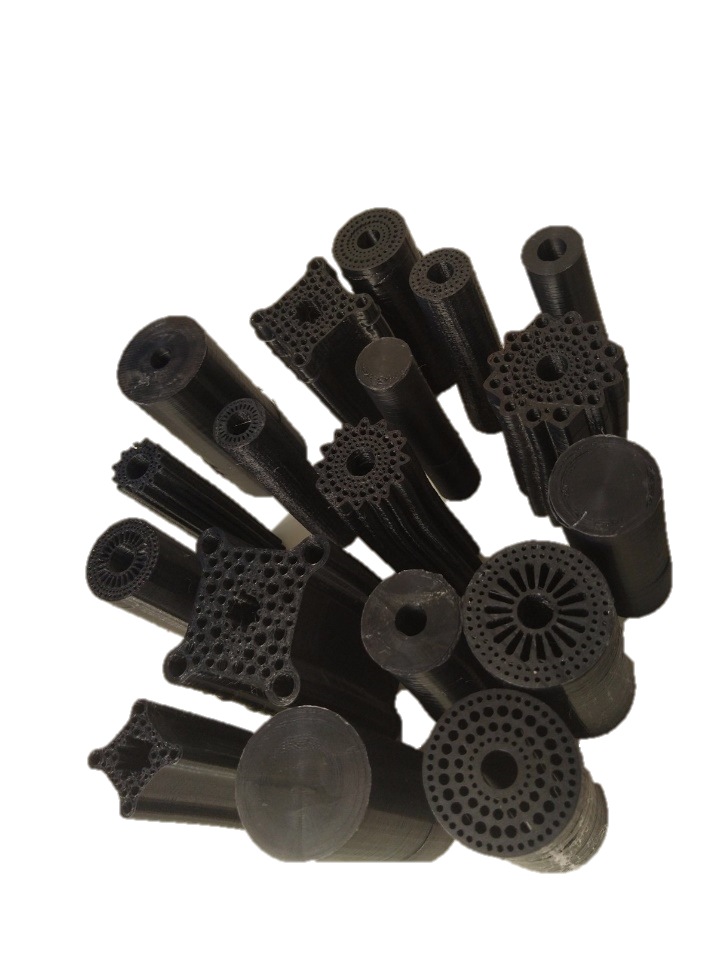
S12. 3D Printed images of biomimetic rods and lattice unit cells with biomimetic rods**


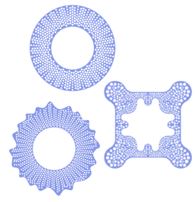


(B)

(A)


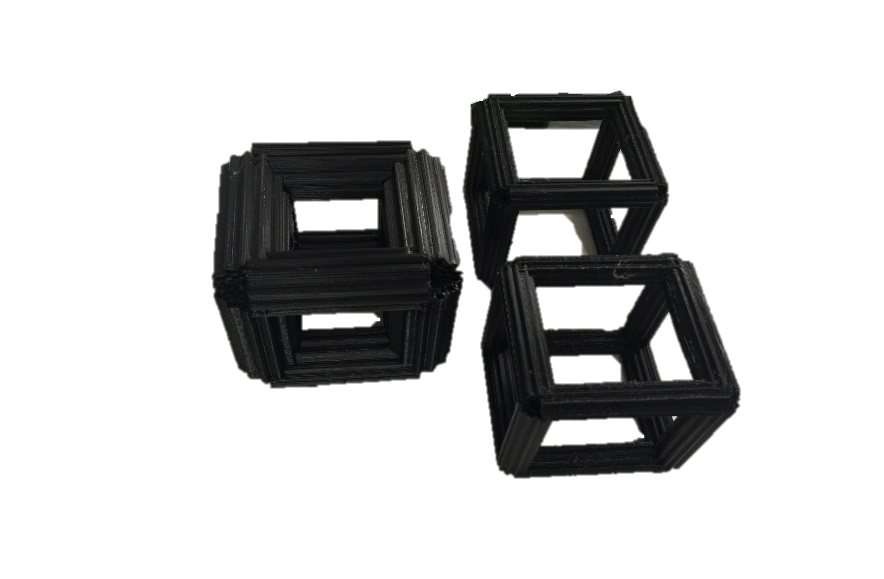


(C)

**Figure S8. (A) 2D representation of biomimetic rod cross-section, (B) 3D Printed biomimetic rods, (C) 3D Printed square lattice unit cells with biomimetic rods**

**S13. Images of optimal lattice unit cells with biomimetic roads using Solidwork design (left), buckling deformation of the corresponding optimal lattice unit cell by ANSYS (right)**

| 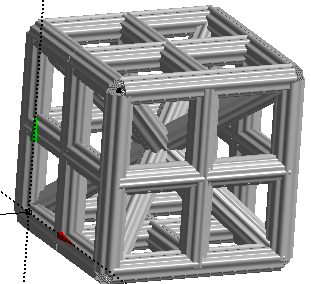 | 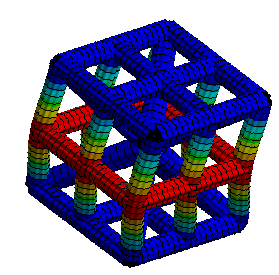 |
| --- | --- |
| 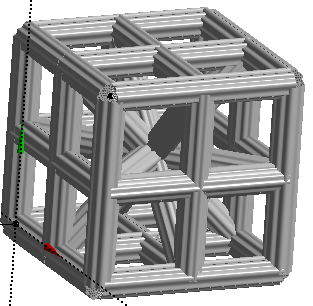 | 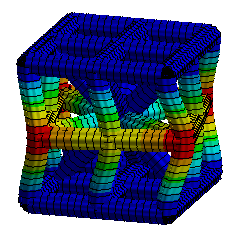 |
| 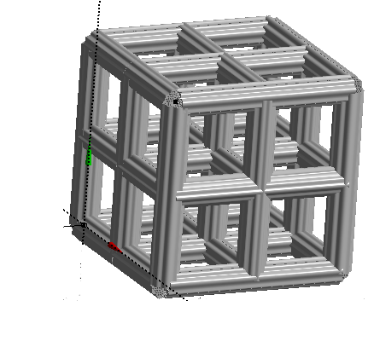 | 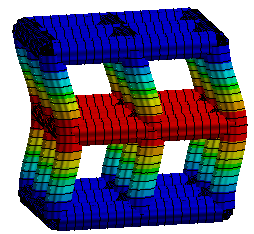 |
| 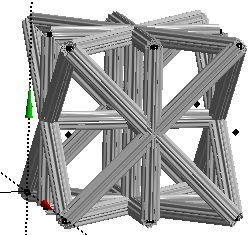 | 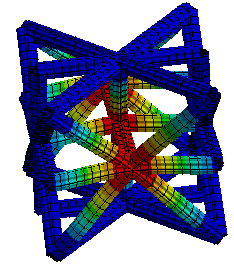 |
| 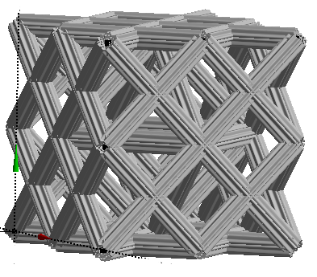 | 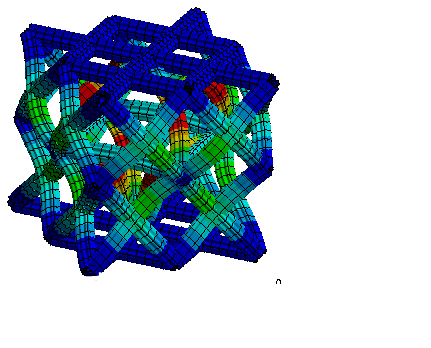 |
| 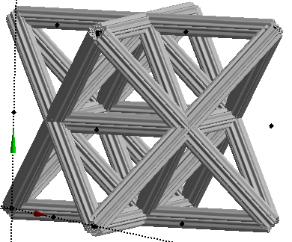 | 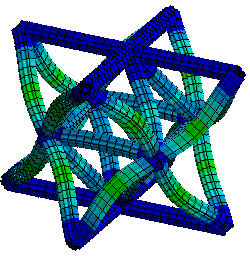 |

**S14. Example optimal lattice unit cells for different deformation criteria**

| Type of load | Best optimal lattice unit cell | % better compared to Octet truss |
| --- | --- | --- |
| Uni-axial compression | 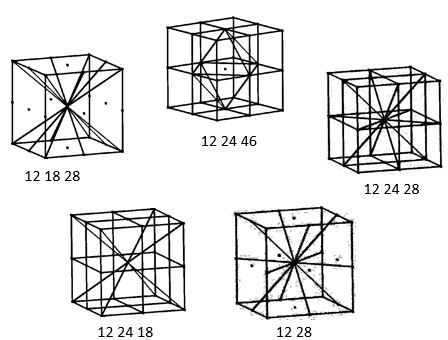12 18 28 | 67% |
| Flexural loading | 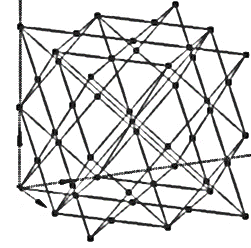16 25 28 | 35% |
| Buckling | 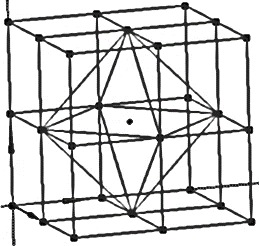  12 24 46 | 160% |
